# Redox-Switchable Naphthalenediimide–NHC Iridium Complexes for Switchable Catalysis in Aniline Methylation with Methanol

**DOI:** 10.1021/acs.inorgchem.5c04216

**Published:** 2025-12-11

**Authors:** Maite Silva-Muñoz, Víctor Martínez-Agramunt, Macarena Poyatos, Eduardo Peris

**Affiliations:** Institute of Advanced Materials (INAM). Centro de Innovación En Química Avanzada (ORFEO−CINQA), 16748 Universitat Jaume I, Av Vicente Sos Baynat S/N, Castellón de la Plana, Castellón E-12071, Spain

## Abstract

We report the synthesis and characterization of rhodium and iridium complexes featuring a naphthalene-diimide (NDI)-functionalized N-heterocyclic carbene (NHC) ligand, in which the NDI unit is directly attached through an imide nitrogen. Electrochemical and spectroelectrochemical studies reveal that one- and two-electron reductions of the NDI moiety moderately enhance the electron-donating ability of the ligand, albeit to a lesser extent than in analogues where the NDI is fused to the carbene backbone. Catalytic investigations demonstrate that the iridium complexes efficiently promote the *N*-methylation of anilines with methanol via a borrowing-hydrogen pathway, outperforming the rhodium analogue. One-electron reduction of the NDI-NHC ligand leads to reversible deactivation of the catalyst, providing direct evidence for redox-switchable control of catalytic activity. Kinetic analyses and substrate studies indicate that imine reduction, rather than methanol dehydrogenation, constitutes the rate-determining step. These findings highlight NDI-NHC ligands as versatile redox-responsive platforms for fine-tuning electronic properties and catalytic performance in hydrgen-borrowing transformations.

## Graphical abstract

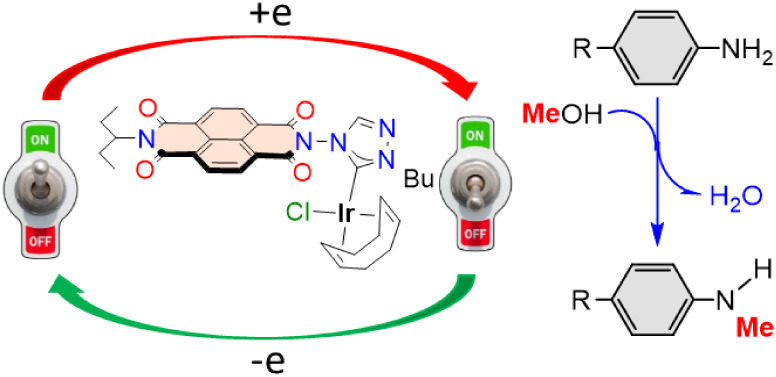

## Introduction

The catalytic *N*-methylation of amines using methanol as a methylating reagent via the borrowing-hydrogen strategy represents one of the simplest and most valuable methods for constructing C–N bonds.^1^ *N*-methylation is widely recognized as a critical chemical transformation due to its significant role in modulating biological activity.^2^ Moreover, the introduction of methyl groups to nitrogen atoms in amine-based pharmaceuticals can improve their pharmacological properties, such as potency, selectivity, and metabolic stability, while also enabling more cost-effective drug synthesis.^3^ Methanol, being an abundant and inexpensive resource, stands out in comparison to other methylating agents such as toxic formaldehyde, methyl iodide, or costly formic acid. Since Grigg and coworkers pioneered the first iridium-catalyzed *N*-methylation of piperazine using methanol, numerous iridium complexes have been developed to promote the alkylation of amines.^4^ Among these, complexes bearing N-heterocyclic carbene (NHC) ligands have gained particular prominence,^5^ especially after Crabtree and coworkers reported in 2015 a series of efficient cationic bis-NHC iridium­(I) catalysts for the selective monomethylation of substituted anilines under microwave irradiation.^5b^ We also contributed to the field several years ago, by reporting how a series of [IrCl_2_Cp*­(NHC)] complexes showed high efficiency in the alkylation of primary amines with primary alcohols.^6^ The mechanism of this reaction is thought to follow the conventional “borrowing hydrogen” pathway. This pathway involves several steps, including alcohol dehydrogenation (resulting in formaldehyde in the case of methanol), metal hydride formation, imine formation, and subsequent reduction of the imine to the final product by the metal hydride.^5d,5e,7^ While the overall reaction pathway is well established, the specific influence of the catalyst’s steric and electronic properties on the efficiency and selectivity of the transformation remains insufficiently understood.

During the past few years, we have been particularly interested in merging the extraordinary photophysical and electrochemical properties of naphthalene-diimides (NDIs)^8^ with those of N-heterocyclic carbene (NHC) ligands. We first designed a series of NDI-NHC ligands, and demonstrated that their corresponding complexes could be successfully employed as redox-switchable catalysts in the cycloisomerization of alkynoic acids^9^ and the hydroamination of acetylenes.^10^ Our studies revealed that the redox-switchable character of the NDI unit allows the modification of the electron-donating strength of the final NDI-NHC ligands in a controlled and reversible manner. Furthermore, merging NHC ligands with the NDI core, allowed three levels of electronic control of the catalyst, given that the ligand can operate either in its neutral form, or in the one- or two-electron reduced forms of the NDI moiety, thus constituting very rare examples of multistate redox switchable catalysis. NDIs are also sensitive to anion−π^11^ and lone pair−π^12^ interactions, because their extraordinary positive quadrupolar moment makes them have a strong tendency to interact with electron-rich lone-pair bearing electronegative atoms. We leveraged these interactions to design halide-sensitive catalysts that could be reversibly tuned between three activity levels by simply adding fluoride,^13^ or chloride.^14^ This led us to introduce the concept of “halide-induced redox-switchable catalysis” (HIRSC) to refer to this effect.^15^ Our studies also provided valuable insights into catalytic mechanisms, offering a straightforward way to investigate how ligand electron-donating properties affect catalytic performance, avoiding the challenges of systematically altering the ligand’s nature, which can lead to misleading results due to changes in steric effects or stability. In these types of NDI-NHC-based metal complexes, the NDI unit of the ligand was incorporated either fused to the NHC ring backbone (Scheme 1, **A**), or as a substituent at the central ring of pincer di-NHC ligands (**B**). We now aim to report a series of rhodium and iridium complexes with NDI-NHC ligands, in which the NDI unit is incorporated as a N-substituent of a triazolylidene ligand (Scheme 1, **C**). This new approach allows us to explore how the electronic properties of the ligand change when the NDI unit is reduced and compare these effects with our earlier examples, where the NDI was fused at the NHC backbone. Our catalytic studies, focused on the methylation of anilines with methanol, provide new insights into how the electron-donating properties of the ligand influence the reaction’s performance.

**Scheme 1:**
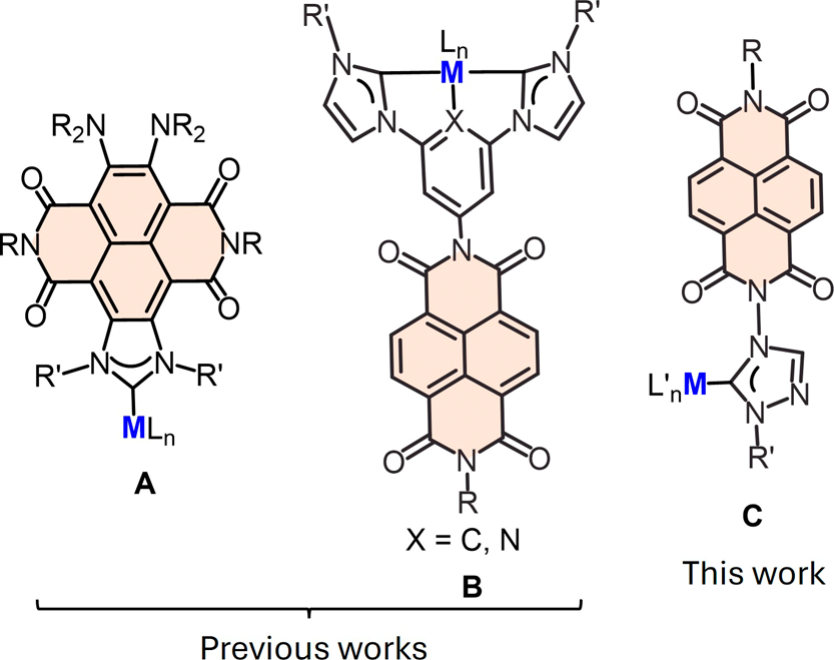
Different Types of NDI-Containing NHC Ligands

## Results and Discussion

The NDI-functionalized triazolium salt [**1**]­(BF_4_) was obtained by condensation of *N*-(1-ethylpropyl)­naphthalene-1,8-naphtalimide-4,5-dicarboxylic anhydride and 4-amino-1,2,4-triazole, and subsequent quaternization with *n*-butyl iodide, which formed [**1**]­(I), as depicted in Scheme 2. Further treatment of [**1**]­(I) with trimethyloxonium tetrafluoroborate allowed the formation of [**1**]­(BF_4_), which was isolated as a pale orange solid. Both [**1**]­(I) and [**1**]­(BF_4_) were characterized by NMR spectroscopy and mass spectrometry (ESI-MS). Then, the rhodium and iridium metal complexes were obtained by reaction of [**1**]­(BF_4_) with [MCl­(COD)]_2_ (M = Rh, Ir) in acetone in the presence of K_2_CO_3_, affording the [MCl­(NDI-NHC)­(COD)] complexes **2** (M = Rh) and **3** (M = Ir), in 72 and 69%, respectively. Both complexes were characterized by means of NMR spectroscopy and mass spectrometry (ESI-MS). The most characteristic resonance on the ^13^C NMR spectra was the one assigned to the metalated carbene carbon, which appeared at 188.2 (d, ^1^ *J*_Rh–C_ = 52.8 Hz) and 183.9 ppm, for **2** and **3**, respectively. The ESI-MS spectra showed base peaks due to [M-Cl]^+^ at *m*/*z* values of 670.1908 and 758.2322, for **2** and **3**, respectively.

**Scheme 2:**
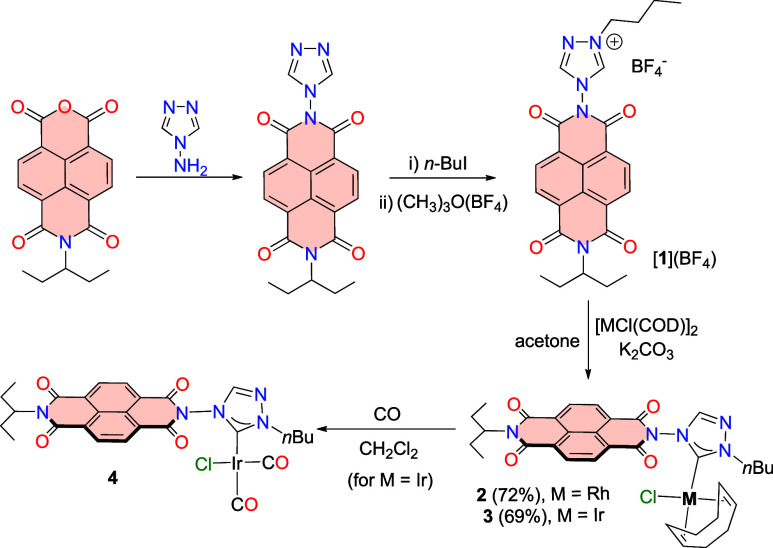
Preparation of the NDI-NHC Rhodium and Iridium Complexes **2**–**4**

The molecular structure of compound **2** was confirmed by single-crystal X-ray diffraction analysis. As shown in Figure 1, the structure comprises a [RhCl­(COD)] fragment (COD = 1,5-cyclooctadiene) coordinated to a 1,2,4-triazolylidene ligand bearing a naphthalene-diimide (NDI) unit and an *n*-butyl group as N-wingtip. The Rh–C_(carbene)_ bond length is 2.023(2) Å. The plane of the naphthalene moiety in the NDI unit is tilted by 75.6° relative to the plane of the triazolylidene ring, while the Rh–Cl bond adopts a quasi-perpendicular orientation (83.3°) with respect to the triazolylidene plane. All other bond lengths and angles fall within expected ranges.

**Figure 1:**
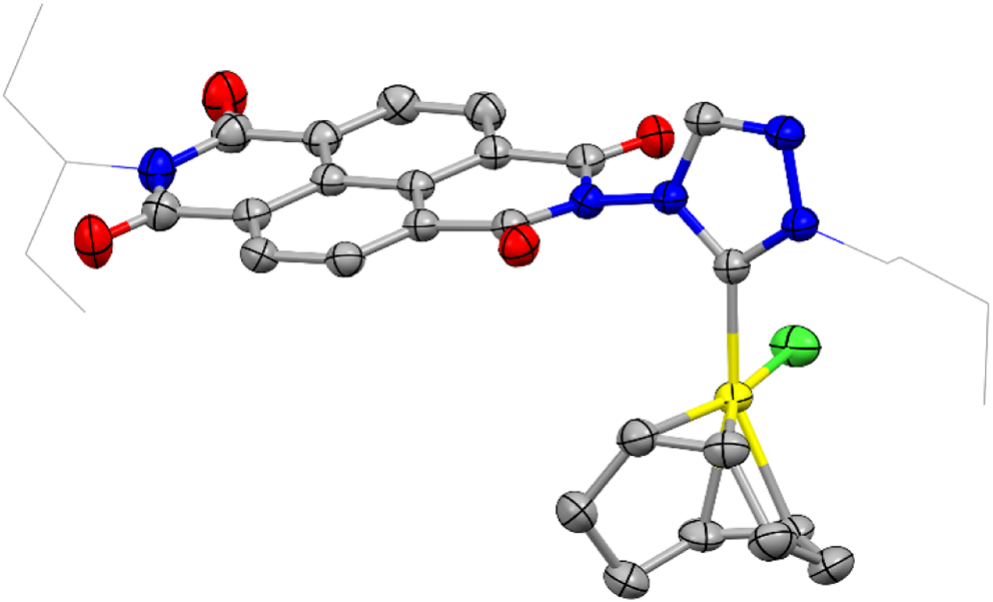
Molecular structure of **2**, obtained from single crystal X-ray diffraction studies. Hydrogen atoms omitted for clarity. Carbon atoms in gray, oxygen atoms in red, nitrogen atoms in light blue, chloride atoms in green and rhodium atom in yellow.

To investigate the electron-donating properties of the new NDI-NHC ligand and how these may be influenced by the reduction of its NDI unit, the iridium carbonyl derivative **4** was synthesized by bubbling CO into a CH_2_Cl_2_ solution of **3** at 0 °C (Scheme 2). The resulting dicarbonylated species was characterized by NMR spectroscopy and ESI-MS. The IR spectrum of **4** reveals two characteristic C–O stretching bands at 2077 and 1995 cm^– 1^. Using the well-established correlation for C–O stretching frequencies,^16^ the estimated Tolman Electronic Parameter (TEP) of this NDI-NHC ligand is calculated to be 2060 cm^– 1^. This value is slightly higher than the TEPs reported for other 1,2,4-triazolylidenes,^17^ indicating that the electron-deficient nature of the NDI unit has an influence on the ligand’s electron-donating ability.

The cyclic voltammograms (CVs) of complexes **2**-**4** (see Figures S24–S29 in the SI) exhibit two well-separated, reversible reduction events (Table 1). For complex **2**, the first reduction occurs at −0.93 V (vs Fc^+^/Fc) and corresponds to the one-electron reduction of the NDI unit within the NDI-NHC ligand, yielding the anionic radical [**2** ^**•**^]**^–^**. The second reduction wave at −1.36 V results in the formation of the dianion [**2**]**^2–^**. The reduction potentials observed for **2**, **3,** and **4** are nearly identical, indicating that the nature of the metal or the substitution of the COD ligand by carbonyls has a negligible effect on the reduction potential of the NDI-decorated NHC ligand. Additionally, the COD-containing complexes **2** and **3** display irreversible oxidation peaks at cathodic potentials (*E*_pc_) of 0.44 and 0.46 V (vs Fc^+^/Fc), respectively, which are attributed to the one-electron oxidation of the metal center. The CV of [**1**]­BF_4_ shows two reversible reduction waves at −0.77 and −1.26 V, corresponding to the sequential one-electron reductions of the NDI unit in the triazolium salt. These values are significantly less negative than those observed for the metal complexes, reflecting the increased ease of reduction due to the positive charge imparted by the triazolium unit, which facilitates electron transfer compared to the neutral complexes **2–4**.

**Table 1:** Electrochemical Properties of Compounds **1–4** ^a^

Compound	*E*_1/2_/V (Δ*E*/mV)	*E*′_1/2_/V (Δ*E*/mV)	*E*″_pc_(V)
**[1](BF_4_)**	–0.77(60)	–1.26(75)	NA
**2**	–0.93(71)	–1.36(85)	0.44
**3**	–0.90(65)	–1.37(77)	0.46
**4**	–0.90(75)	–1.36(81)	NA

a Cyclic voltammograms performed in dry CH_2_Cl_2_ with 1 mM analyte and 0.25 M [N­(*n*Bu)_4_]­[PF_6_]. Measurements performed at 100 mV s^–1^ and referenced vs ferrocenium/ferrocene.

UV–vis Spectroelectrochemical (SEC) studies were conducted to investigate the nature and stability of the species formed upon the one- and two-electron reductions of the NDI unit in the complexes. These experiments were carried out using an Optically Transparent Thin Layer Electrochemical (OTTLE) cell in CH_2_Cl_2_, with progressively more negative potentials applied while monitoring the corresponding UV–vis spectra. The resulting spectral series for the iridium complex **3** are shown in Figure 2. The UV–vis spectrum of complex **3** exhibits a vibronically resolved band with a peak at 350 nm (solid blue line in Figure 2), which is attributed to transitions centered around the NDI core. As the reduction progresses, the intensity of this band diminishes, accompanied by the emergence of broad new bands at 475, 720, and 775 nm (solid green line), corresponding to the formation of the [**3** ^•^]^−^ species. Further reduction results in the disappearance of these bands and the appearance of new peaks at 420, 565, and 625 nm (solid orange line), which are associated with the two-electron reduced species, [**3**]^2–^. Throughout these reduction steps, no intermediate species were observed, as evidenced by isosbestic points at 375, 455, and 540 nm. Upon application of positive potentials, the neutral form of complex **3** was fully restored, confirming that both reduction processes are fully reversible and that all species involved in these redox events are stable under the experimental conditions used to carry out the SEC experiment.

**Figure 2:**
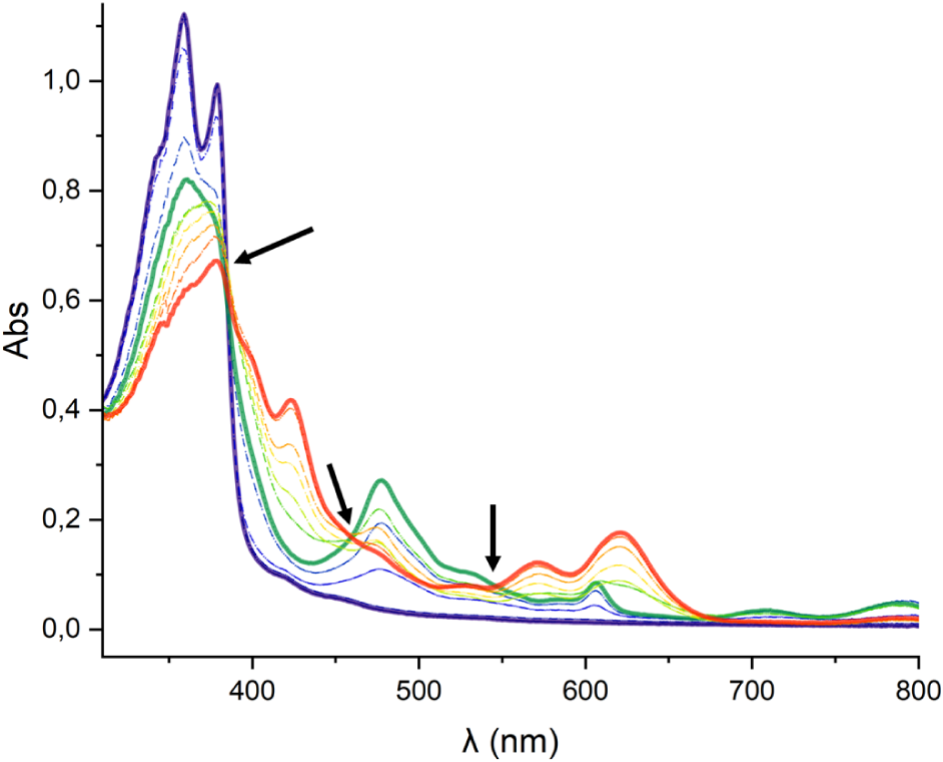
UV–vis SEC monitoring reduction of **3** in dry CH_2_Cl_2_ (0.25 M [N­(*n*Bu)_4_]­[PF_6_]). The electrochemical reduction was performed applying progressively lower potentials with a Pt working electrode, Pt counter-electrode, and Ag wire pseudoreference electrode. The solid lines represent the spectra of the starting (blue), singly reduced (green) and doubly reduced (orange) species. Arrows are used to signal the isosbestic points.

We also investigated the chemical reduction of complexes **2** and **3** using cobaltocene. The addition of one equivalent of Cp_2_Co to a solution of either complex resulted in the complete disappearance of the NDI signals in the ^1^H NMR spectra (see Figures S32 and S33 in the SI). As pointed out in previous studies,^18^ these spectral changes are consistent with the formation of a radical anion, with the unpaired electron localized on the NDI core. Addition of further equivalents of Cp_2_Co produced no additional spectral changes, indicating that cobaltocene promotes only a single-electron reduction of the complexes. Subsequent treatment of a 1:1 mixture of **2** (or **3**) and Cp_2_Co with acetylferrocenium tetrafluoroborate restored the ^1^H NMR signals of the neutral complexes, demonstrating that the redox process is chemically reversible. These observations are significant for understanding the redox-switchable behavior exhibited by complex **3** in the catalytic reactions discussed below.

As observed in previous studies of **A**-type complexes (Scheme 1), where the NDI unit is fused to the backbone of the NHC ligand, the reduction of the NDI moiety significantly enhances the electron-donating character of the ligand. In this context, we aimed to explore whether a similar effect occurs in our new NDI-NHC ligand, where the NDI is bound to the nitrogen of the triazolylidene. To investigate this, we performed an IR-SEC experiment on the iridium–carbonyl complex **4**. Figure 3 presents the IR spectra obtained during the progressive reduction of complex **4** in CH_2_Cl_2_. Upon one-electron reduction, the two C–O stretching bands at 2077 and 1995 cm^–1^ (solid blue line in Figure 3) disappear, and two new C–O bands at 2072 and 1993 cm^–1^ (solid green line) appear. These changes correspond to the formation of the one-electron reduced species, [**4** ^•^]^−^. Further reduction, achieved by applying a more negative potential, leads to the disappearance of the bands at 2072 and 1993 cm^–1^, with the concomitant appearance of new bands at 2068 and 1988 cm^–1^ (solid orange line), which are attributed to the two-electron reduced species, [**4**]^2–^. This indicates that the first reduction results in an average shift Δν­(CO) of 3.5 cm^–1^, while the second reduction produces a further shift of 4.5 cm^–1^, demonstrating that the ligand’s electron-donating power can be subtly tuned in a two-step manner. Importantly, the effect of NDI reduction in complex **4** is notably weaker than in complexes where the NDI unit is fused directly to the NHC backbone (**A**, Scheme 1, or **6** in Chart [Fig chart1]), for which each single-electron reduction of the NDI unit causes a decrease in ν­(CO) by approximately 10 cm^–1^ (and about 20 cm^–1^ for the two-electron reduction) in the related [IrCl­(NDI-NHC)­(CO)_2_] complexes.^9a,10^ This significant difference highlights a key observation: substituents directly bound to the *sp* ^2^ carbon atoms of the NHC ligand exert a much stronger effect on the electronic properties of the ligand than those bound to the nitrogen atom. These findings suggest that modifying the electronic nature of the N-wingtips can finely tune the electron-donating power of the NHC ligand, enabling more precise modulation of the complex’s reactivity and catalytic activity.

**Figure 3:**
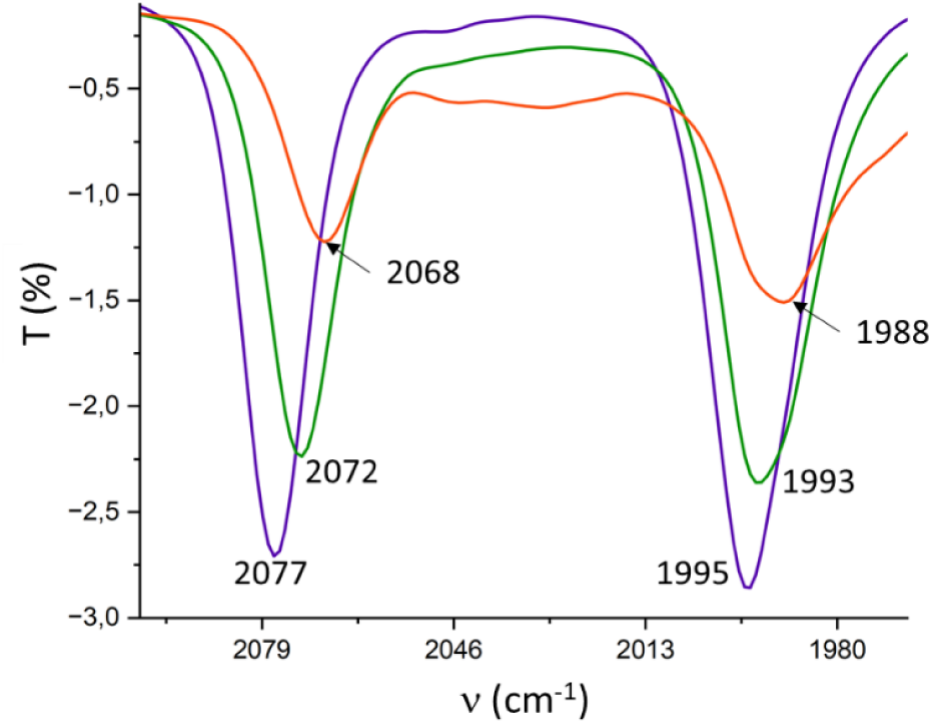
IR SEC monitoring reduction of **4** in dry CH_2_Cl_2_ (0.25 M [N­(*n*Bu)_4_]­[PF_6_]). The electrochemical reduction was performed applying progressively lower potentials with a Pt working electrode, Pt counter-electrode, and Ag wire pseudoreference electrode. The solid lines represent the IR spectra of **4** (blue), [**4** ^•^]^−^. (green) and [**4**]^2–^ (orange) species. Wavenumbers in cm^–1^ are shown for each of the bands.

**1 chart1:**
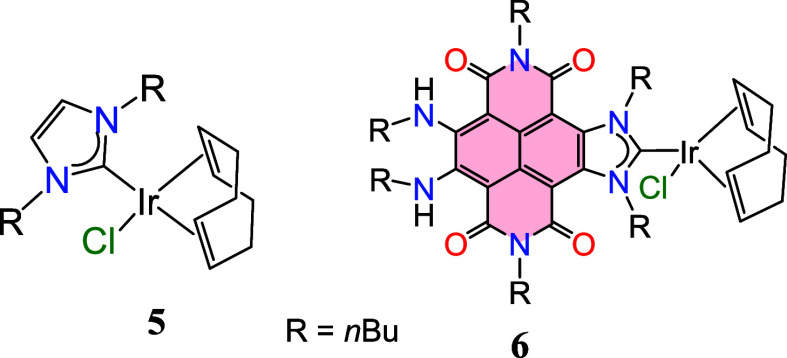
Complexes **5** and **6**

To explore the catalytic activities of complexes **2–4**, we focused on the methylation of primary amines using methanol as the reagent. As previously noted, while the mechanism for this transformation is well-established, the precise influence of the catalyst’s steric and electronic properties on the efficiency and selectivity of the process is not fully understood. In this context, we hypothesized that incorporating the NDI moiety as a redox-sensitive tag into our complexes could provide valuable insight into how variations in the electron-donating character of the NDI–NHC ligand influence catalytic performance. For comparison, we also carried out reactions using [IrCl_2_Cp*]_2_, [IrCl­(COD)]_2_, [IrCl­(InBu)­(COD)] (**5**, InBu = 1,3-di-*n*-butylimidazol-2-ylidene), and the iridium­(I) complex **6**, which we reported previously (Chart [Fig chart1]).^9a^ Complexes [IrCl_2_Cp*]_2_,^19^ [IrCl­(COD)]_2_,^5d^ were included because they have been employed in prior studies on amine methylation. The Ir–NHC complexes **5** and **6** were examined due to their electron-donating properties, which are comparable to those of the monoreduced form of **3** (3^•–^). Their catalytic behavior thus should provide a useful benchmark for assessing how the electronic nature of the ligand affects the catalytic performance of the iridium complexes.

Reactions were conducted in a high-pressure Schlenk tube at 150 °C in methanol, using aniline as model substrate. The results, summarized in Table 2, reveal that when aniline was used as the standard substrate, the iridium catalysts (complexes **3** and **4**) outperformed the rhodium catalyst (complex **2**) in terms of activity (compare entries 1 and 2 with entries 6 and 7). Specifically, the rhodium catalyst produced only 5% *N*-methylaniline after 4 h, despite using double the catalyst loading (1 mol %) compared to the iridium catalysts (0.5 mol %). This outcome aligns with our expectations, as iridium complexes are generally known to exhibit superior catalytic activity compared to analogous rhodium complexes for this type of reaction. Among the iridium catalysts, the COD-containing complex **3** demonstrated higher activity than the iridium dicarbonyl complex **4** (compare entries 1 and 3 with entries 6 and 7). Catalyst **4** also outperformed catalysts **5** and **6**, suggesting that the slightly stronger electron-donating character of the NHC ligands in the latter may be detrimental to this particular catalytic transformation. Similarly, both [IrCl_2_Cp*]_2_ and [IrCl­(COD)]_2_ exhibited very low activity under the applied reaction conditions, affording maximum product yields of 49% and 25%, respectively, after 5 h of reaction.

**Table 2:**
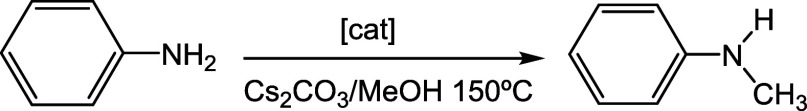
*N*-Methylation of Aniline with Methanol Using Catalyst **2**–**6** ^a^

Entry	Catalyst	Time (min)	Yield (%)
1^b^	**2**	240	5
2	**3**	60	51
3	**3**	120	89
4	**3** + 0.6 mol % [CoCp_2_]	60	5
5	**3** + 0.6 mol % [CoCp_2_]	120	7
6^c^	**4**	60	30
7^c^	**4**	120	60
8	**5**	60	9
9	**5**	180	34
10	**5**	300	48
11	**6**	60	4
12	**6**	120	12
13	**6**	300	18
14	[IrCl_2_Cp*]_2_	60	4
15	[IrCl_2_Cp*]_2_	180	32
16	[IrCl_2_Cp*]_2_	300	49
17	[IrCl(COD)]_2_	60	8
18	[IrCl(COD)]_2_	180	18
19	[IrCl(COD)]_2_	300	25

a Reaction conditions: Reactions carried out in a high-pressure Schlenk tube loaded with 1.5 mL of MeOH, 0.5 mmol of aniline, and 0.25 mmol of Cs_2_CO_3_, and 0.5 mol % of catalyst at 150 °C.

b Catalyst loading = 1 mol %.

c Same reaction conditions with addition of 0.6 mol % of cobaltocene. All product yields were calculated using gas chromatography using 1,3,5-trimethoxybenzene (0.5 equiv) as internal standard.

Additionally, we sought to determine whether the reduction of the NDI moiety in the catalyst would influence the reaction outcome. To achieve this, we used cobaltocene to carry out a one-electron reduction of the NDI moiety on the NDI-NHC ligand, as described in our previous studies.^9,10,20^ With a redox potential of −1.33 V (vs Fc^+^/Fc),^21^ cobaltocene is a suitable additive for the one-electron reduction of our Ir-NDI-NHC catalyst, since the first reduction potential of **3** is −0.90 V. The data in Table 2 reveal that the addition of cobaltocene effectively quenched the activity of catalyst **3** in the methylation of aniline, resulting in only 7% product yield after 2 h (see entry 5).

Since as shown in Table 2 complex **3** was the most efficient catalyst, it was selected for further studies using 4-methyl-aniline, 4-nitro-aniline, 4-fluoro-aniline, cyclohexylamine, and 3-pentamine as substrates. As shown in Table 3, the efficiency of the methylation process varied significantly with the nature of the substrates, with the product yields of the aromatic amines being significantly higher than those shown by the two aliphatic ones. Among the aromatic amines, we observed that product yields decreased in the order: 4-nitroaniline > aniline > 4-methyl-aniline > 4-fluoroaniline.

**Table 3:** *N*-Methylation of Primary Amines with Methanol Using **3** as Catalyst^a^

Entry	Substrate	Time (min)	Yield (%)
1	4-methylaniline	60	42
2		120	77
3	4-nitroaniline	60	87
4		120	99
5	4-fluoroaniline	60	48
6		120	75
7	cyclohexylamine	60	2
8		180	20
9		300	28
10	3-pentamine	60	10
11		180	21
12		300	30

a Reaction conditions: Reactions carried out in a high-pressure Schlenk tube loaded with 1.5 mL of MeOH, 0.5 mmol of amine, and 0.25 mmol of Cs_2_CO_3_ and 0.5 mol % of catalyst at 150 °C. All product yields were calculated using gas chromatography using 1,3,5-trimethoxybenzene (0.5 equiv) as internal standard.

Next, we conducted kinetic studies to determine the rate order with respect to the catalyst. For this, we employed the variable determination analysis, which involves visually comparing the normalized concentration profiles at different reaction times.^22^ Catalytic reactions were monitored using three distinct concentrations of catalyst **3** (0.2, 0.5, and 1 mol %). To minimize potential errors arising from opening the reaction vessels, we conducted parallel sets of identical experiments. As shown in Figure 4a, the reactions followed pseudo-first-order kinetics with respect to the substrate. The normalized concentration profiles in Figure 4b are consistent with a first-order dependence on catalyst **3**. This observed rate order supports the conclusion that the reaction proceeds via homogeneous catalysis, even under the harsh reaction conditions employed. Additional normalized profiles corresponding to hypothetical 0.5 and second-order dependencies on the catalyst are provided in Figure S35 of the Supporting Information file for comparison.

**Figure 4:**
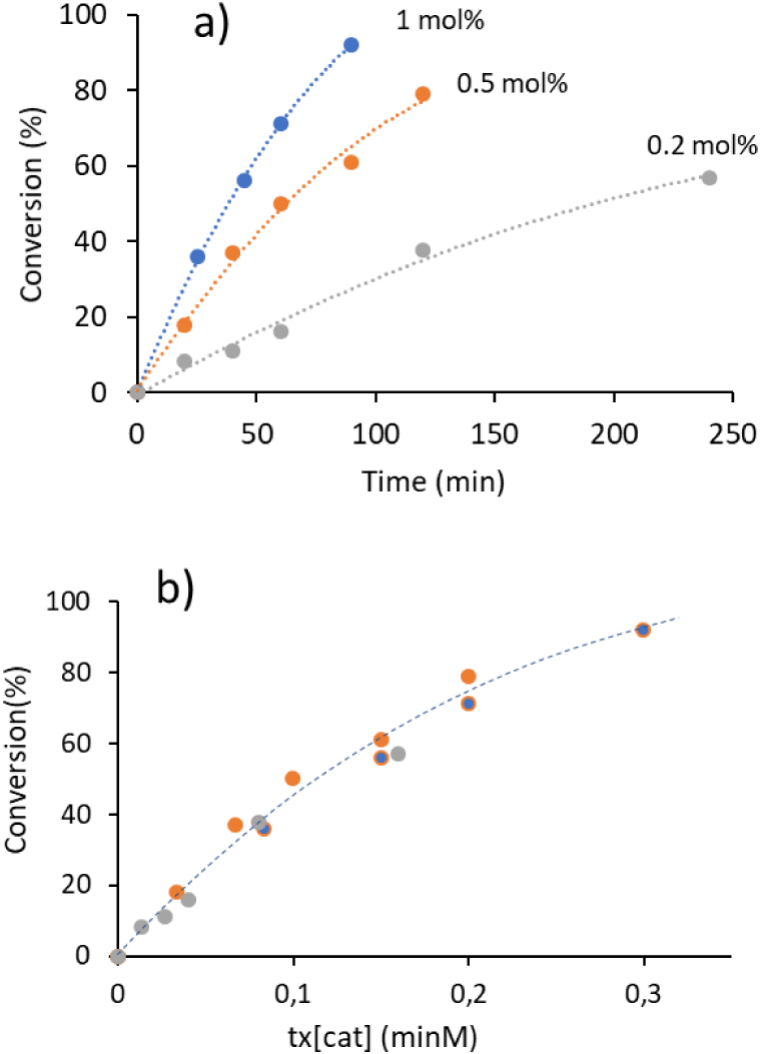
(a) Time dependent profile of the methylation of aniline at three different concentrations of catalyst **3** (0.2, 0.5, and 1 mol %). (b) Normalized profile assuming a reaction order of 1 with respect to the catalyst. All reactions were carried out in a high-pressure Schlenk tube loaded with 1.5 mL of MeOH, 0.5 mmol of aniline, and 0.25 mmol of Cs_2_CO_3_. Conversions calculated using gas chromatography using 1,3,5-trimethoxybenzene (0.5 equiv) as internal standard.

To further investigate the impact of the electron-donating process on the reaction, we aimed to toggle between the active and inactive forms of the catalyst during the course of the reaction (Figure 5). Initially, we allowed the reaction to progress for 60 min at 150 °C in the presence of catalyst **3** (0.5 mol %) and cobaltocene (0.6 mol %), and observed that only 5% of the product had been formed. Next, we introduced acetylferrocenium tetrafluoroborate ([Fe­(Ν^5^-C_5_H_4_COCH_3_)­Cp]­(BF_4_); 0.6 mol %) to oxidize the catalyst back to its active form, **3**, and continued the reaction for an additional 60 min. After this, the product yield had increased to 31% *N*-methylamine. Then, we added cobaltocene again and monitored the reaction for another 60 min, during which the product yield rose to 35%. This demonstrates that during the reaction periods when the catalyst was in its inactive form ([**3** ^•^]^−^), only 5% of the product was formed after 1 h. In contrast, when the catalyst was active (in the second period, catalyzed by **3**), the product formation increased by 26%. This result is significant because it suggests that while cobaltocene quenches the activity of the catalyst, the catalyst’s activity can be restored upon the addition of an oxidant. This strongly implies that cobaltocene does not decompose the catalyst but rather transforms it into a dormant form. However, it is important to note that the catalyst’s activity after reactivation with acetylferrocenium tetrafluoroborate (26% yield) was notably lower than the initial activity of **3** under standard conditions (51%, as shown in Table 1, entry 2). This reduction in activity can be attributed to the adverse effects of opening the reaction vessel to add the reducing and oxidizing agents, as indicated by the experimental setup in Figure 5.

**Figure 5:**
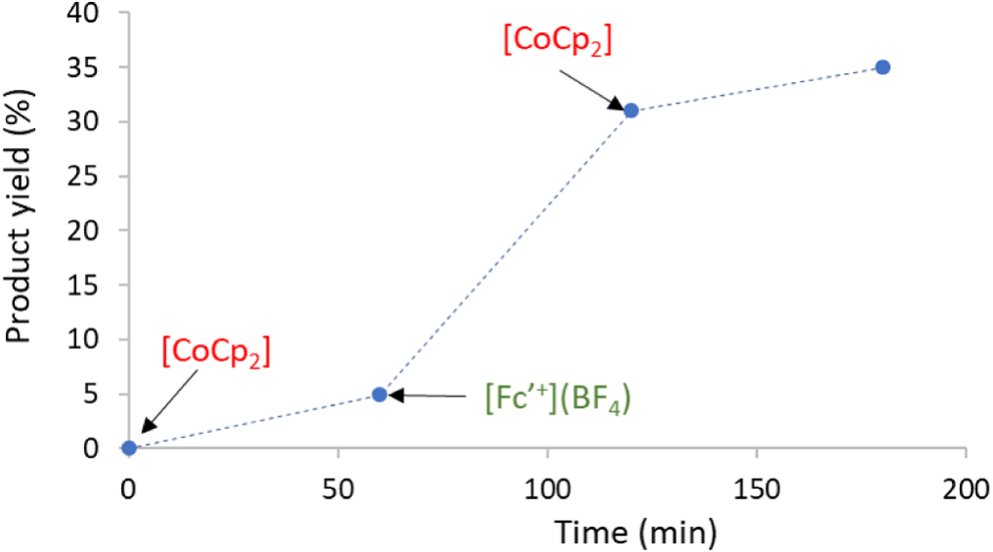
Reaction profile of the methylation of aniline using catalyst **3**, after subsequent additions of 1.2 mol % of cobaltocene ([CoCp_2_]), acetylferrocenium tetrafluoroborate ([Fc’^+^]­(BF_4_)) and cobaltocene. The reaction was performed using 0.5 mol % of **3** at 150 °C. Product yields were calculated by GC using 1,3,5-trimethoxybenzene as standard. The results shown are the average of two independent experiments.

The widely accepted mechanism for the methylation of anilines with methanol involves a tandem sequence consisting of three key steps (Figure 6). First, methanol undergoes dehydrogenation to generate formaldehyde and an iridium­(I) hydride species (Step 1). This is followed by condensation of the amine with formaldehyde to form an imine, which then inserts into the Ir–H bond to yield an amido intermediate (Step 2). Finally, methanolysis of the Ir–N bond leads to the formation of the methylated amine and regeneration of the catalyst (Step 3). The reaction is intrinsically challenging due to the presence of two opposing, mirror-image steps: methanol dehydrogenation and imine hydrogenation. While previous studies have proposed that the rate-determining step is the initial dehydrogenation of methanol and formation of the Ir–H intermediate (Step 1),^5d^ our findings instead suggest that the rate-limiting steps are associated with imine reduction; namely, steps 2 and 3. This conclusion is supported by two key observations: (1) the reaction proceeds faster with anilines bearing electron-withdrawing substituents, which are more prone to dissociation; and (2) increasing the electron-donating character of the NDI–NHC ligand leads to a decrease in reaction rate. The latter effect would be expected to promote imine insertion into the Ir–H bond (Step 2), rather than facilitate β-hydride elimination during methanol dehydrogenation (Step 1). As an alternative explanation, the slower reaction rate observed for the reduced form of the iridium catalyst could arise from electrostatic repulsion between the anionic (reduced) catalyst, generated upon addition of cobaltocene, and the methoxide anion (MeO^–^), produced by reduction of methanol with the base. This repulsion would hinder formation of the Ir-OMe complex that initiates the catalytic cycle. However, the observation that the neutral [IrCl­(NHC)­(COD)] complexes **5** and **6**-bearing NHC ligands with electron-donating properties comparable to those of the reduced NDI–NHC ligand in **3**- exhibit negligible activity in the *N*-methylation of aniline suggests that the change in catalytic activity is primarily due to the altered electron-donating character of the ligand, rather than electrostatic repulsion between the anionic catalyst and the methoxide anion. Overall, these results are consistent with prior mechanistic studies on the alkylation of amines with primary alcohols, further supporting a rate-limiting role for imine reduction in this system.^23^

**Figure 6:**
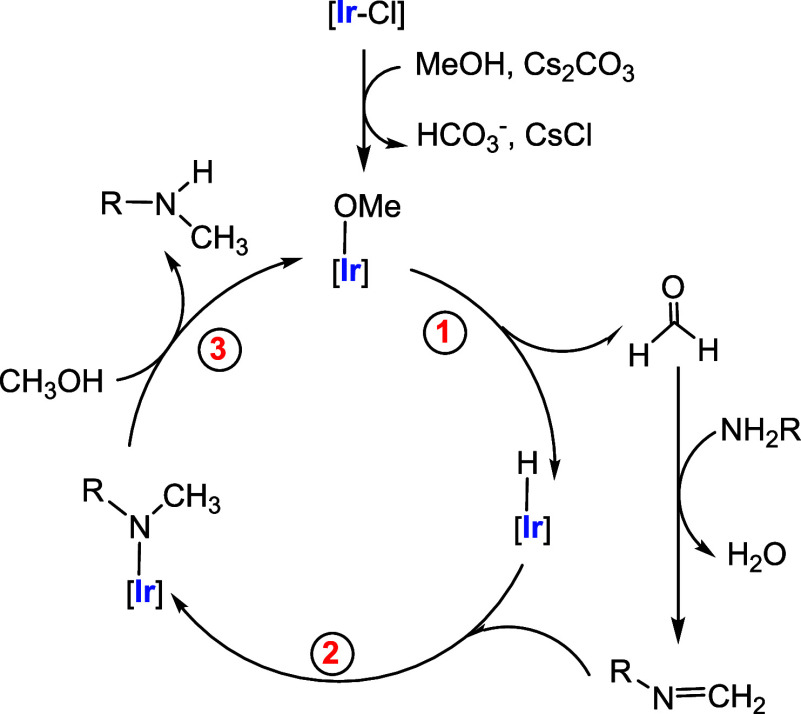
Mechanism proposed for the iridium-catalyzed methylation of anilines using methanol.

## Conclusions

In summary, we have synthesized a series of rhodium and iridium complexes featuring a naphthalene-diimide (NDI)-functionalized N-heterocyclic carbene (NHC) ligand, in which the NDI unit is directly connected to the NHC through one of its imide nitrogen atoms. The influence of NDI reduction on the electron-donating properties of the ligand was evaluated using IR spectroelectrochemical studies. These revealed that one- and two-electron reductions of the NDI moiety result in a modest but non-negligible increase in the ligand’s donor strength. This effect is notably less pronounced than in related systems where the NDI unit is directly fused to the NHC backbone. The catalytic implications of this tunable donor strength were investigated in the context of aniline methylation using methanol. Our results show that one-electron reduction of the NDI-NHC ligand leads to a significant decrease in the activity of the iridium catalyst, an effect that is reversible upon reoxidation to the neutral state. Although detailed mechanistic studies were not undertaken, this reversible deactivation, along with preliminary kinetic analysis, suggests that the rate-determining step likely involves imine reduction rather than methanol dehydrogenation. Overall, this work demonstrates the potential of NDI-NHC complexes as redox-switchable catalysts, offering not only tunable activity but also valuable mechanistic insight into catalytic processes.

## Experimental Section

Anhydrous solvents were dried using a solvent purification system (SPS M BRAUN) or purchased and degassed prior to use by purging them with dry nitrogen. All the other reagents were used as received from the commercial suppliers. Column chromatography was performed using silica gel (60–120 mesh). NMR spectra recorded on a Bruker 400 or 300 MHz using CDCl_3_, CD_2_Cl_2_ or DMSO-*d*_6_ as solvents, chemical shifts (δ) are expressed in ppm using the residual proton resonance of the solvent as an internal standard. All coupling constants (*J*) are expressed in hertz (Hz). High-resolution mass spectra (HRMS) were recorded on a Micromass Quatro LC instrument; nitrogen was employed as drying and nebulizing gas. Infrared spectra (FTIR) were performed on a Bruker Equinox 55 spectrometer with a spectral window of 4000–400 cm^–1^. UV–visible absorption spectra were recorded on a Varian Cary 300 BIO spectrophotometer under ambient conditions. Elemental analyses were carried out on a LECO TruSpec Micro Series. To determine the bulk purity of the complexes, their ^1^H NMR spectra were recorded in the presence of an equimolar amount of 1,3,5-trimethoxybenzene as an internal standard. The bulk purity was determined prior to each catalytic test and used to accurately calculate the catalyst loading.

### Synthesis and Characterization of the Compounds


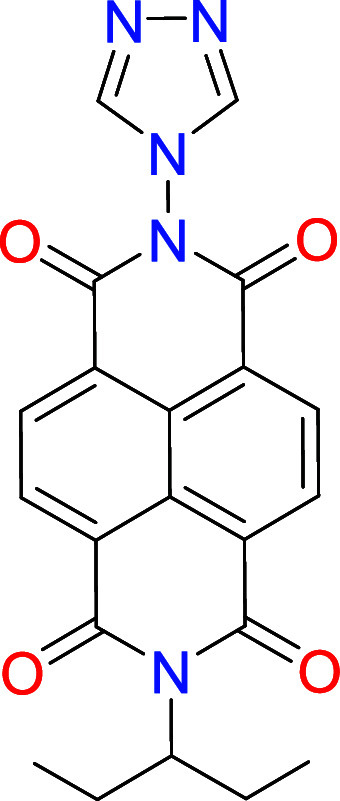


### Synthesis and Characterization of **I**

A high pressure Schlenk tube fitted with a Teflon cap was equipped with *N*-(1-ethylpropyl)­naphthalene-1,8-naphthalimide-4,5-dicarboxylic anhydride (1.58 g, 4.68 mmol). The solid was suspended in dry DMF (7 mL) and 4-amino-1,2,4-triazole (393 mg, 4.68 mmol) was then added. The resulting suspension was heated at 140 °C for 20 h. After that, with the suspension still hot, it was transferred to a 500 mL Erlenmeyer flask and 100 mL of H_2_O were added. The resulting precipitate was collected via filtration and dried under vacuum. Compound **I** was isolated as a white solid in 94% yield (1774.3 mg). ^1^H NMR (300 MHz, CDCl_3_): δ = 8.90–8.82 (m, 4H, C*H*_NDI_), 8.35 (s, 2H, C*H*_triazole_), 5.10–4.98 (m, 1H, C*H*(CH_2_CH_3_)_2_), 2.33–2.14 (m, 2H, CH­(C*H*_2_CH_3_)_2_), 2.05–1.86 (m, 2H, CH­(C*H*_2_CH_3_)_2_), 0.92 (t, ^3^ *J*_H–H_ = 7.1 Hz, 6H, CH­(CH_2_C*H*_3_)_2_). ^13^C­{^1^H} NMR (75 MHz, CDCl_3_): δ = 160.1 (*C*=O_NDI_), 142.3 (*C*H_triazole_), 133.0 (*C*H_NDI_), 131.4 (*C*H_NDI_), 127.3 (*C*_NDI_), 126.7 (*C*_NDI_), 124.8 (*C*_NDI_), 58.8 (*C*H­(CH_2_CH_3_)_2_), 25.0 (CH­(*C*H_2_CH_3_)_2_), 11.5 (CH­(CH_2_ *C*H_3_)_2_). HRMS (20 V, *m*/*z*): 404.1364 [M + H]^+^. (Calcd for [M + H]^+^: 404.1359).

### Synthesis and Characterization of **[1]­(I)**


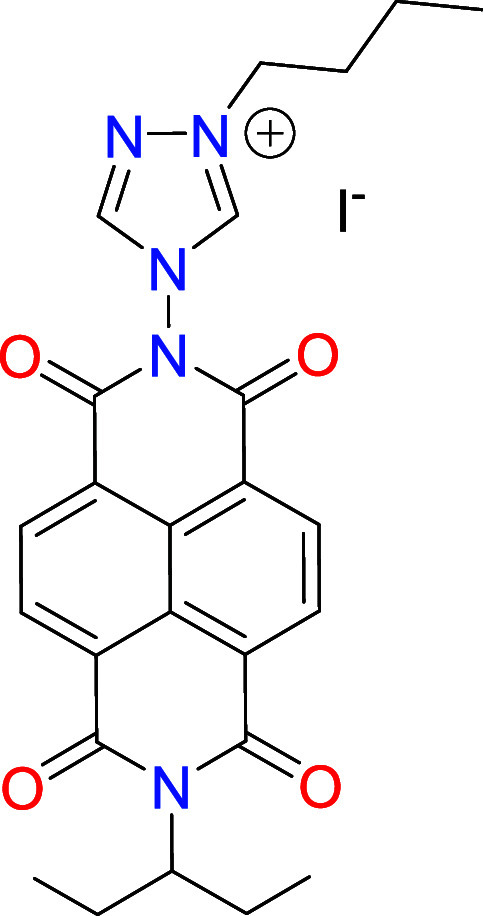


Compound **I** (1 g, 2.48 mmol) was placed in a high-pressure Schlenk tube fitted with a Teflon cap. An excess of iodobutane (5 mL) was added and the mixture was allowed to stir for 72 h at 140 °C under N_2._ After this time, diethyl ether was added, and the resulting solid was collected by filtration. Compound [**1**]­(I) was isolated as a red orange solid in 89% yield (1.20 g). ^1^H NMR (300 MHz, DMSO-*d*_6_): δ = 10.69 (s, 1H, NC*H*N), 9.67 (s, 1H, C*H*_triazole_), 8.89–8.77 (m, 4H, C*H*_NDI_), 4.91–4.87 (m, 1H, C*H*(CH_2_CH_3_)_2_), 4.69 (t, ^3^ *J*_H–H_ = 7 Hz, 2H, NC*H*_2_CH_2_CH_2_CH_3_), 2.20–2.10 (m, 2H, CH­(C*H*_2_CH_3_)_2_), 2.01–1.86 (m, 4H; 2H, CH­(C*H*_2_CH_3_)_2_ and 2H, NCH_2_C*H*_2_CH_2_CH_3_), 1.36 (q, ^3^ *J*_H–H_ = 7.5 Hz, 2H, NCH_2_CH_2_C*H*_2_CH_3_), 0.97 (t, ^3^ *J*_H–H_ = 7.5 Hz, 3H, NCH_2_CH_2_CH_2_C*H*_3_), 0.87 (t, ^3^ *J*_H–H_ = 7.5 Hz, 6H, CH­(CH_2_C*H*_3_)_2_)). ^13^C­{^1^H} NMR (75 MHz, DMSO-*d*_6_): δ = 159.7 (*C*=O_NDI_), 145.5 (N*C*HN), 144.3 (*C*H_triazole_), 132.1 (*C*H_NDI_), 130.9 (*C*H_*NDI*_), 126.7 (*C*_NDI_), 126.1 (*C*_NDI_), 124.7 (*C*_NDI_), 57.6 (*C*H­(CH_2_CH_3_)_2_), 52.9 (N*C*H_2_CH_2_CH_2_CH_3_), 29.8 (NCH_2_ *C*H_2_CH_2_CH_3_), 24.4 (CH­(*C*H_2_CH_3_)_2_), 18.7 (NCH_2_CH_2_ *C*H_2_CH_3_), 13.2 (NCH_2_CH_2_CH_2_ *C*H_3_), 11.3 (CH­(CH_2_ *C*H_3_)_2_). HRMS (20 V, *m*/*z*): 460.1987 [M]^+^. (Calcd for [M]^+^: 460.1985).

### Synthesis and Characterization of Compound **[1]­(BF**_**4**_)


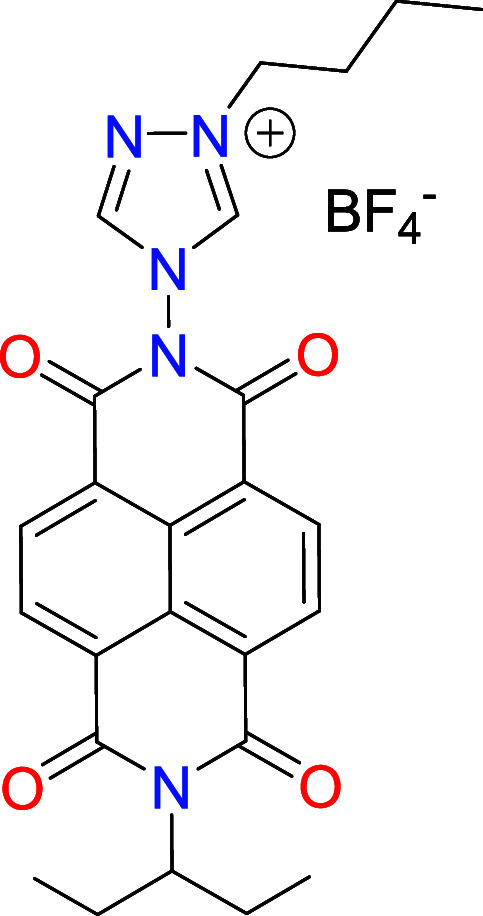


Compound [**1**]­(I) (300 mg, 0.51 mmol) and trimethyloxonium tetrafluoroborate (120 mg, 0.81 mmol) were placed together in a Schlenk tube. The Schlenk tube was evacuated and filled with nitrogen three times. Dry CH_2_Cl_2_ (15 mL) was added, and the mixture was allowed to stir overnight at room temperature. The volume was reduced by half under reduced pressure and the solid formed was collected by filtration after the addition of diethyl ether. Compound [**1**]­(BF_4_) was isolated as a pale orange solid in 65% yield (180 mg). ^1^H NMR (300 MHz, DMSO-*d*_6_): δ = 10.68 (s, 1H, NC*H*N), 9.66 (s, 1H, C*H*_triazole_), 8.94–8.75 (m, 4H, C*H*_NDI_), 4.97–4.84 (m, 1H, C*H*(CH_2_CH_3_)_2_), 4.69 (t, ^3^ *J*_H–H_ = 7.0 Hz, NC*H*_2_CH_2_CH_2_CH_3_), 2.24–2.06 (m, 2H, CH­(C*H*_2_CH_3_)_2_), 2.04–1.83 (m, 4H; 2H CH­(C*H*_2_CH_3_)_2_ and 2H, NCH_2_C*H*_2_CH_2_CH_3_), 1.36 (sext, ^3^ *J*_H–H_ = 7.4 Hz, 2H, NCH_2_CH_2_C*H*_2_CH_3_), 0.97 (t, ^3^ *J*_H–H_ = 7.4 Hz, 3H, NCH_2_CH_2_CH_2_C*H*_3_), 0.87 (t, ^3^ *J*_H–H_ = 7.4 Hz, 6H, CH­(CH_2_C*H*_3_)_2_). ^13^C­{^1^H} NMR (75 MHz, DMSO-*d*_6_): δ = 162.9 (*C*=O_NDI_), 159.8 (*C*=O_NDI_), 145.5 (*C*H_triazole_), 143.3 (*C*H_triazole_), 132.1 (*C*H_NDI_), 130.9 (*C*H_NDI_), 128.1 (*C*_NDI_), 126.8 (*C*_NDI_), 126.1 (*C*_NDI_), 124.7 (*C*_NDI_), 57.6 (*C*H­(CH_2_CH_3_)_2_), 52.9 (N*C*H_2_CH_2_CH_2_CH_3_), 29.8 (NCH_2_ *C*H_2_CH_2_CH_3_), 24.4 (CH­(*C*H_2_CH_3_)_2_). 18.7 (NCH_2_CH_2_ *C*H_2_CH_3_), 13.2 (NCH_2_CH_2_ *C*H_2_CH_3_), 11.3 (CH­(CH_2_ *C*H_3_)_2_). HRMS (20 V, *m*/*z*): 460.1988 [M]^+^. (Calcd for [M]^+^: 460.1985).

### Synthesis and Characterization of **2**


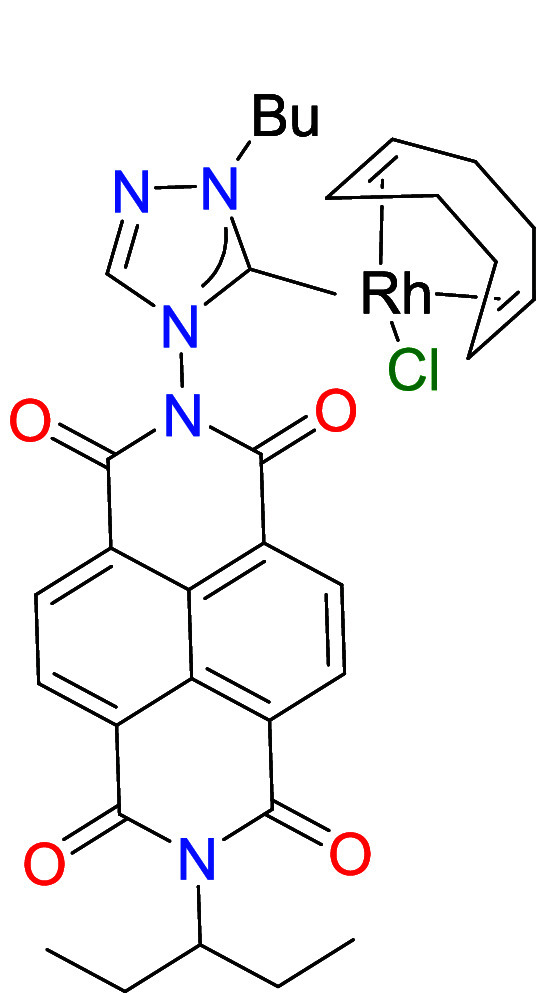


A Schlenk flask was loaded with compound [**1**]­(BF_4_) (250 mg, 0.46 mmol), [RhCl­(COD)]_2_ (120 mg, 0.24 mmol) and K_2_CO_3_ (100 mg, 0.72 mmol). The mixture was suspended in acetone (20 mL) and stirred for 12 h at room temperature. The crude mixture was filtered through Celite pad. The resulting solution was concentrated under reduced pressure and then precipitated by the addition of pentane. Purification was carried out by column chromatography on silica gel using dichloromethane and acetone as the eluent. The eluting solvent was initially dichloromethane to remove the excess of the unreacted rhodium dimer, then the desired complex was eluded with a 9:1 mixture of dichloromethane/acetone. Complex **2** was isolated as a brown solid in 72% yield (230 mg). ^1^H NMR (400 MHz, CDCl_3_): δ = 8.95–8.78 (m, 4H, C*H*_NDI_), 8.16 (s, 1H, C*H*_triazole_), 5.10–5.00 (m, 1H, C*H*(CH_2_CH_3_)_2_), 4.98–4.46 (m, 4H; 2H, C*H*_COD_ and 2H, NC*H*_2_CH_2_CH_2_CH_3_), 3.58 (br s, 2H, C*H*_COD_), 2.56–1.64 (m, 14H; 4H, CH­(C*H*_2_CH_3_)_2_ and 8H, C*H*_2 COD_ and 2H, NCH_2_C*H*_2_CH_2_CH_3_), 1.53 (sext, ^3^ *J*_H–H_ = 7.4 Hz, 2H, NCH_2_CH_2_C*H*_2_CH_3_), 1.07 (t, ^3^ *J*_H–H_ = 7.3 Hz, 3H, NCH_2_CH_2_CH_2_C*H*_3_), 0.94 (t, ^3^ *J*_H–H_ = 7.4, 6H, CH­(CH_2_C*H*_3_)_2_). ^13^C­{^1^H} NMR (400 MHz, CDCl_3_): δ = 188.2 (d, ^1^ *J*_Rh–C_ = 52.8 Hz, Rh-*C*_carbene_), 163.1 (*C*=O_NDI_), 160.4 (*C*=O_NDI_), 143.5 (*C*H_triazole_), 132.0 (*C*H_NDI_), 131.6 (*C*H_NDI_), 128.4 (*C*_NDI_), 127.5 (*C*_NDI_), 126.8 (*C*_NDI_), 125.6 (*C*_NDI_), 100.0 (d, ^1^ *J*_Rh–C_ *=* 20.2 Hz, Rh-*C*H_COD_), 70.5 (Rh-*C*H_COD_), 58.7 (*C*H­(CH_2_CH_3_)_2_), 53.7 (N*C*H_2_CH_2_CH_2_CH_3_), 32.9 (*C*H_2 COD_), 32.0 (*C*H_2 COD_), 28.7 (NCH_2_ *C*H_2_CH_2_CH_3_), 25.1 (CH­(C*H*_2_ *C*H_3_)_2_), 19.9 (NCH_2_CH_2_ *C*H_2_ *C*H_3_), 13.9 (NCH_2_CH_2_CH_2_ *C*H_3_), 11.5 (CH­(CH_2_ *C*H_3_)_2_). HRMS (20 V, *m*/*z*): 670.1908 [M-Cl]^+^. (Calcd for [M-Cl]^+^: 670.1901).

### Synthesis and Characterization of **3**


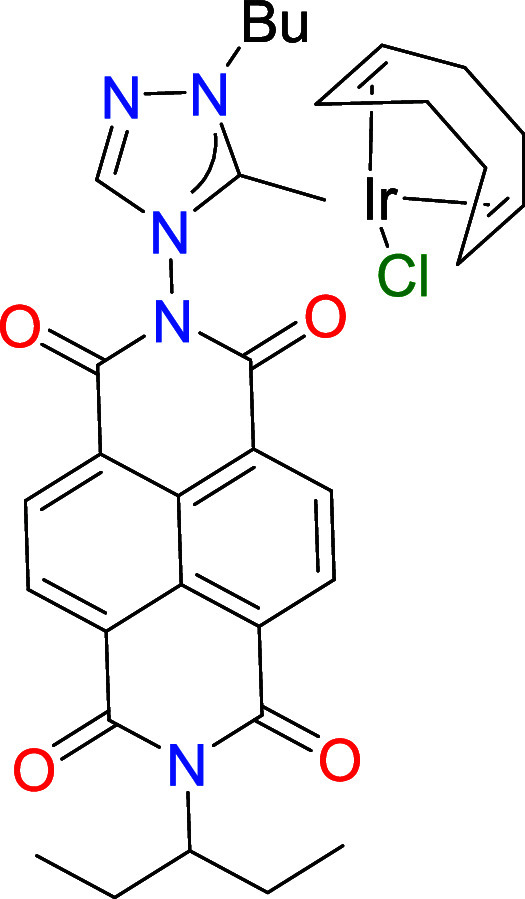


Complex **3** was prepared following the general procedure employed to prepare complex **2**, by reacting [**1**]­(BF_4_) (350 mg, 0.64 mmol), K_2_CO_3_ (132 mg, 0.96 mmol) and [IrCl­(COD)]_2_ (214 mg, 0.32 mmol). Complex **3** was isolated as brown solid in 69% yield (351 mg). ^1^H NMR (300 MHz, CDCl_3_): δ = 8.94–8.73 (m, 4H, C*H*_NDI_), 8.15 (s, 1H, C*H*_triazole_), 5.12–4.98 (m, 1H, C*H*(CH_2_CH_3_)_2_), 4.68–4.41 (m, 3H; 1H, C*H*_COD_ and 2H, NC*H*_2_CH_2_CH_2_CH_3_), 4.23 (br s, 1H, C*H*_COD_), 3.16 (br s, 2H, C*H*_COD_), 2.29–2.14 (m, 6H; 2H, CH­(C*H*_2_CH_3_)_2_ and 2H, C*H*_2 COD_ and 2H, NCH_2_C*H*_2_CH_2_CH_3_), 2.04–1.86 (m, 4H; 2H, CH­(C*H*_2_CH_3_)_2_ and 2H, C*H*_2 COD_), 1.57–1.40 (m, 6H; 2H, NCH_2_CH_2_C*H*_2_CH_3_ and 4H, C*H*_2 COD_), 1.04 (t, ^3^ *J*_H–H_ = 7.3 Hz, 3H, NCH_2_CH_2_CH_2_C*H*_3_), 0.94 (t, ^3^ *J*_H–H_ = 7.4 Hz, 6H, CH­(CH_2_C*H*_3_)_2_).^13^C­{^1^H} NMR (75 MHz, CDCl_3_): δ = 183.9 (Ir-*C*_carbene_), 142.0 (*C*H_triazole_), 126.3 (*C*_NDI_), 125.6 (*C*_NDI_), 124.3 (*C*_NDI_), 86.9 (*C*H_COD_), 57.5 (*C*H­(CH_2_CH_3_)_2_), 53.2 (*C*H_COD_), 52.2 (N*C*H_2_CH_2_CH_2_CH_3_), 32.5 (*C*H_2 COD_) 30.8 (NCH_2_ *C*H_2_CH_2_CH_3_), 28.1 (*C*H_2 COD_), 23.9 (CH­(*C*H_2_CH_3_)_2_), 18.7 (NCH_2_CH_2_ *C*H_2_CH_3_), 12.7 (NCH_2_CH_2_CH_2_ *C*H_3_), 10.3 (CH­(CH_2_ *C*H_3_)_2_). HRMS (20 V, *m*/*z*): 758.2322 [M-Cl]^+^, 801.2740 [M-Cl+CH_3_CN]^+^. (Calcd for [M-Cl]^+^: 760.2475; Calcd for [M-Cl+CHCN]^+^: 802.2818).

### Synthesis and Characterization of **4**


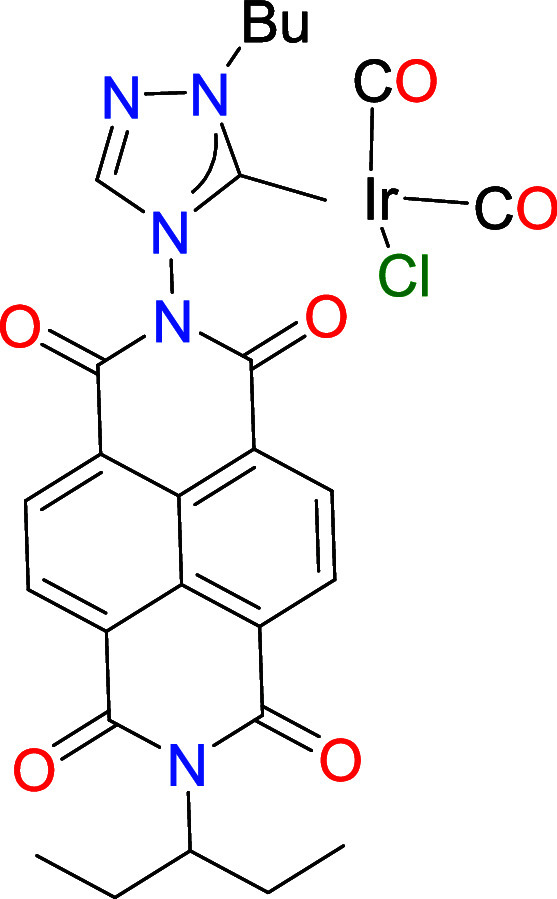


Complex **3** (50 mg, 0.071 mmol) was placed in a two-neck round-bottom flask and dissolved in CH_2_Cl_2_ (20 mL). The solution was cooled at 0 °C with an ice bath. Then, CO (g) was bubbled for 15 min. A change in color was observed from brown to yellow after 10 min. The solution was then concentrated under reduced pressure, and, after the addition of pentane, the compound was collected via filtration as a dark yellow solid in 81% yield (80 mg). ^1^H NMR (400 MHz, CD_2_Cl_2_): δ = 8.89–8.80 (m, 4H, C*H*_NDI_), 8.39 (s, 1H, C*H*_triazole_), 5.07–4.98 (m, 1H, C*H*(CH_2_CH_3_)_2_), 4.59 (t, ^3^ *J*_H–H_ = 7.3 Hz, 2H, NC*H*_2_CH_2_CH_2_CH_3_), 2.28–2.15 (m, 2H, CH­(C*H*_2_CH_3_)_2_), 2.12–2.03 (m, 2H, NCH_2_C*H*_2_CH_2_CH_3_), 2.00–1.88 (m, 2H, CH­(C*H*_2_CH_3_)_2_), 1.53–1.42 (m, 2H, NCH_2_CH_2_C*H*_2_CH_3_), 1.03 (t, ^3^ *J*_H–H_ = 7.3 Hz, NCH_2_CH_2_CH_2_C*H*_3_), 0.91 (t, ^3^ *J*_H–H_ = 7.5 Hz, 6H, CH­(CH_2_C*H*_3_)_2_). ^13^C­{^1^H} NMR (101 MHz, CD_2_Cl_2_): δ = 180.2 (Ir-*C*_carbene_), 179.3 (Ir-*C*O), 167.4 (Ir-*C*O), 160.4 (*C*=O_NDI_), 143.8 (*C*H_*triazole*_), 133.1 (*C*H_NDI_), 131.5 (*C*H_NDI_), 129.1­(*C*_NDI_), 127.2 (*C*_NDI_), 125.1 (*C*_NDI_), 58.9 (*C*H­(CH_2_CH_3_)_2_), 54.6 (N*C*H_2_CH_2_CH_2_CH_3_), 31.8 (NCH_2_ *C*H_2_CH_2_CH_3_), 25.4 (CH­(*C*H_2_CH_3_)_2_), 20.0 (NCH_2_CH_2_ *C*H_2_CH_3_), 13.8 (NCH_2_CH_2_CH_2_ *C*H_3_), 11.5 (CH­(CH_2_ *C*H_3_)_2_). FT-IR (CH_2_Cl_2_): 1995 and 2077 cm^–1^ (ν Ir-CO) cm^–1^. HRMS (20 V, *m*/*z*): 708.1442 [M-Cl]^+^. (Calcd for [M-Cl]^+^: 708.1434).

## Supplementary Material



## Notes

Additional experimental details, materials, and methods, NMR, UV–vis and emission spectra, electrochemical, spectroelectrochemical, and X-ray diffraction studies (PDF)

## Abbreviations

NDI: naphthalene-diimide
NHC: N-heterocyclic carbene
TBACl: tetrabutylammonium chloride
