# Supplementary material for: Redox-Switchable Naphthalenediimide–NHC Iridium Complexes for Switchable Catalysis in Aniline Methylation with Methanol
Source: Inorg Chem. 2025 Dec 11;64(51):25151–60. doi: 10.1021/acs.inorgchem.5c04216 (PMC12754753; doi:10.1021/acs.inorgchem.5c04216)
Supplement: Supplementary file 1 [file ic5c04216_si_001.pdf]

Supporting Information

Redox-switchable naphthalenediimide–NHC Iridium Complexes for Switchable Catalysis in Aniline Methylation with Methanol

Maite Silva-Muñoz, Víctor Martínez-Agramunt, Macarena Poyatos and Eduardo Peris**

Institute of Advanced Materials (INAM). Centro de Innovación en Química Avanzada (ORFEO-CINQA). Universitat Jaume I. Av. Vicente Sos Baynat s/n. Castellón. E-12071. Spain. Email: poyatosd@uji.es, eperis@uji.es

1. Spectroscopic data **S2-S10**

1.1 ^{1}H and $^{13}C\{^{1}H\}$ NMR spectra of **I** S2

1.2 ^{1}H and $^{13}C\{^{1}H\}$ NMR spectra of [**1**](I) S3

1.3 ^{1}H, $^{13}C\{^{1}H\}$ and HSQC NMR spectra of [**1**](BF_4) S4

1.4. ^{1}H, $^{13}C\{^{1}H\}$ and HSQC NMR spectra of **2** S6

1.5. ^{1}H, $^{13}C\{^{1}H\}$ and HSQC NMR spectra of **3** S8

1.6. ^{1}H and $^{13}C\{^{1}H\}$ NMR spectra of **4** S10

2. HRMS spectra of the compounds **S12-S12**

3. X-ray crystallography **S13**

4. Electrochemical studies **S14-S17**

4.1. Electrochemical measurements S14

4.2. Spectroelectrochemical studies S16

5. Chemical reduction of complexes 2 and 3 with cobaltocene **S18**

6. Catalytic studies **S19-S21**

6.1. N-methylation of primary amines using methanol S19

6.2. Determination of the reaction order with respect to catalyst **3** S22

6.3. Redox switching experiments S23

7. References S23

1. Spectroscopic data

1.1. ^{1}H and ^{13}C{^{1}H} NMR spectra of I

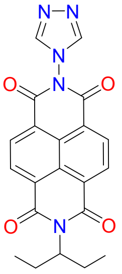

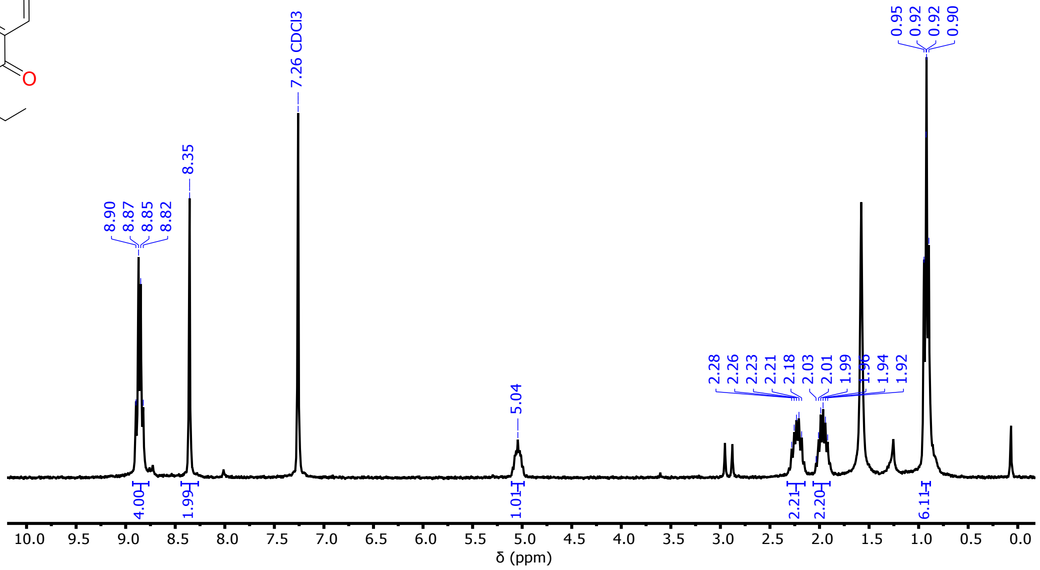

Figure S1. ^{1}H NMR spectrum (300 MHz, $CDCl_3$) of **I**

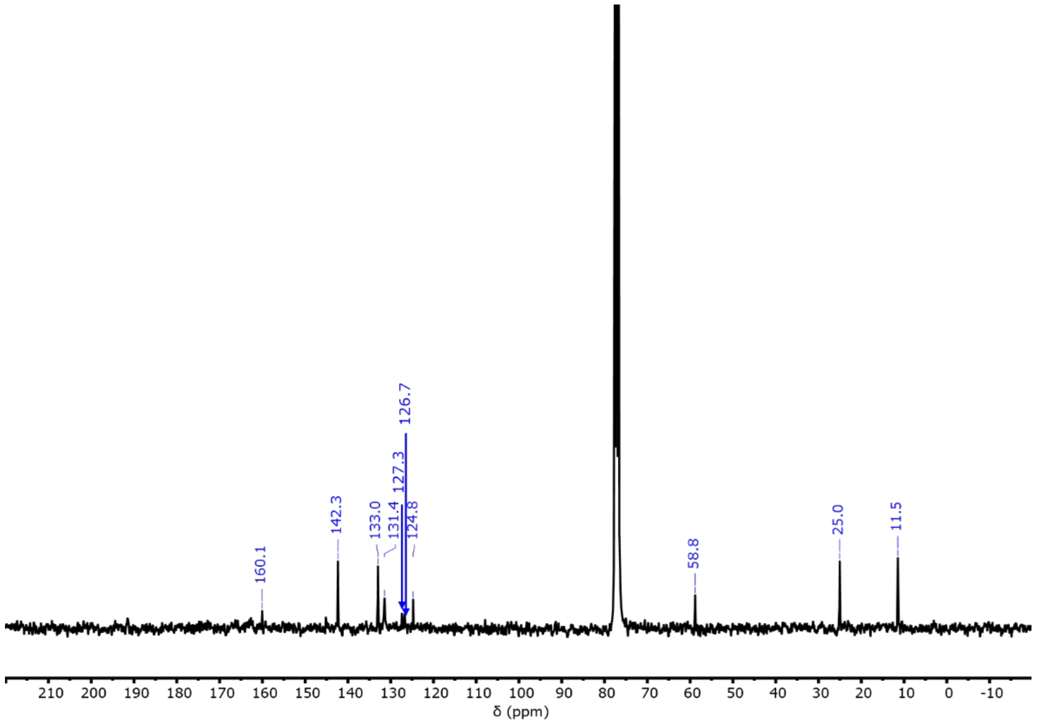

Figure S2. ^{13}C{^{1}H} NMR spectrum (75 MHz, $CDCl_3$) of **I**

1.2. ^{1}H and ^{13}C{^{1}H} NMR spectra of [1](I)

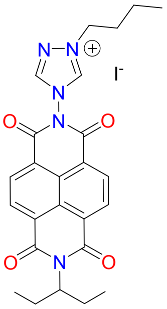

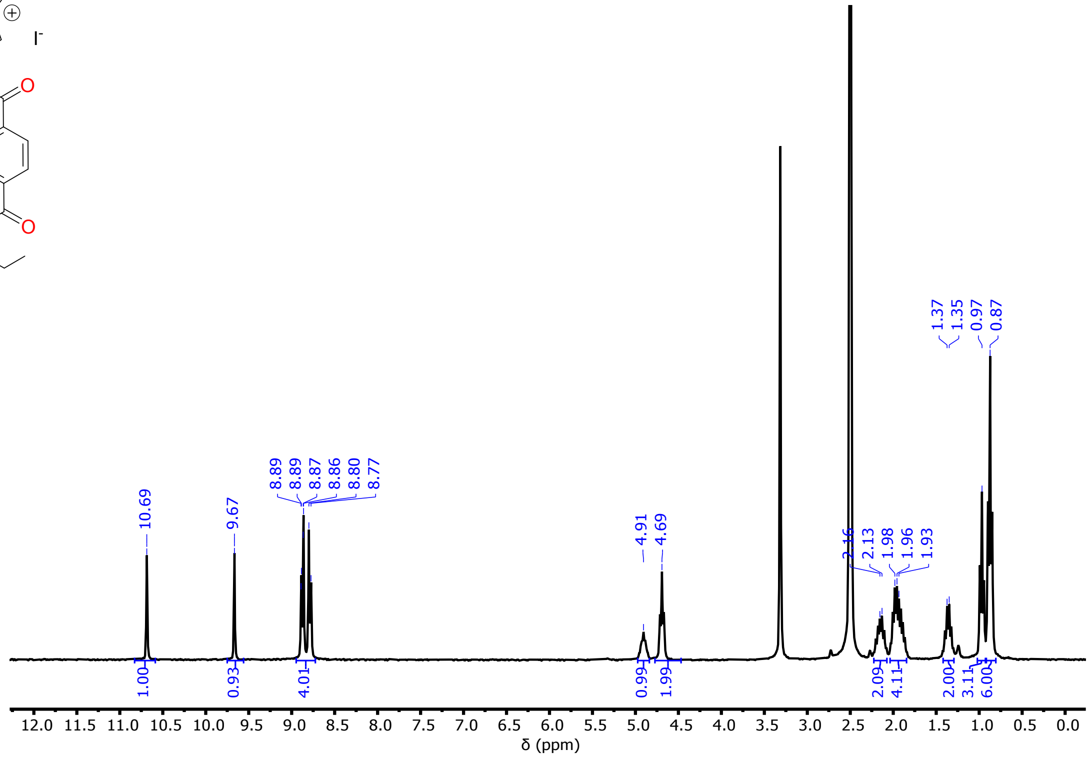

Figure S3. ^{1}H NMR spectrum (300 MHz, DMSO-d_6) of [**1**](I)

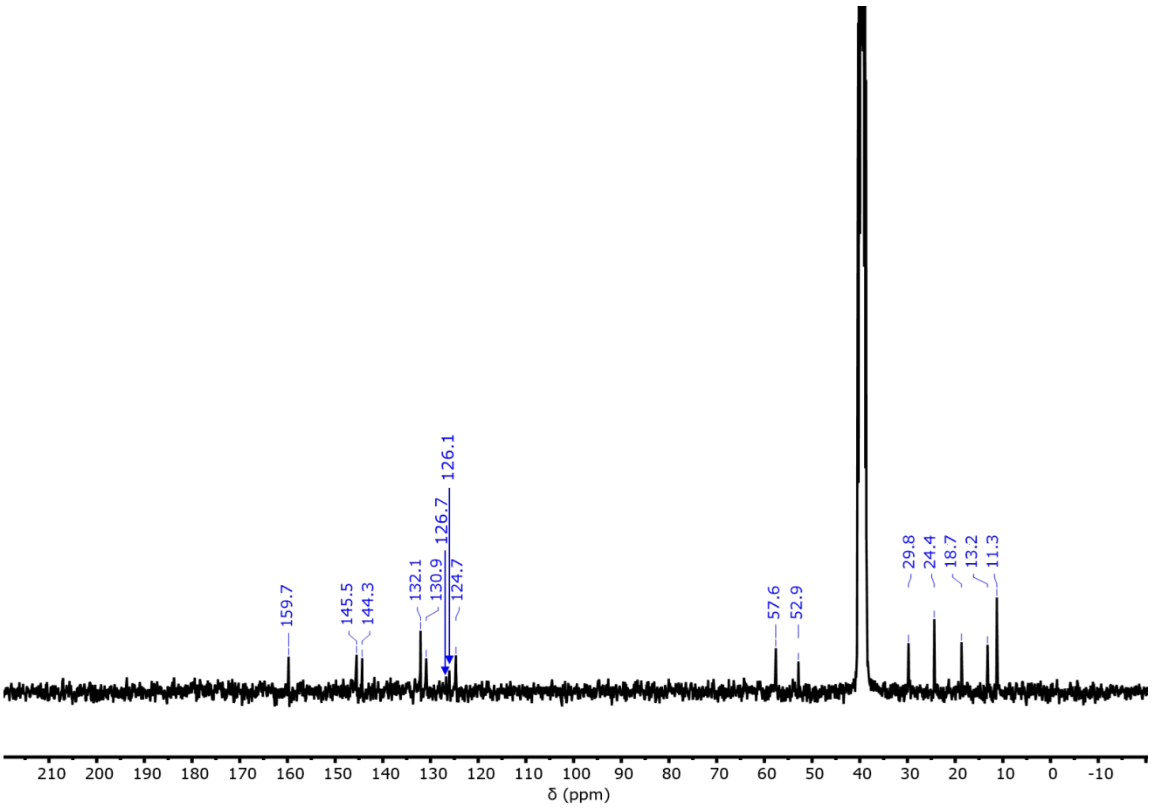

Figure S4. ^{13}C{^{1}H} spectrum (75 MHz, DMSO-d_6) of [**1**](I)

1.3. ^{1}H, $^{13}C\{^{1}H\}$ and HSQC NMR spectra of [1](BF_4)

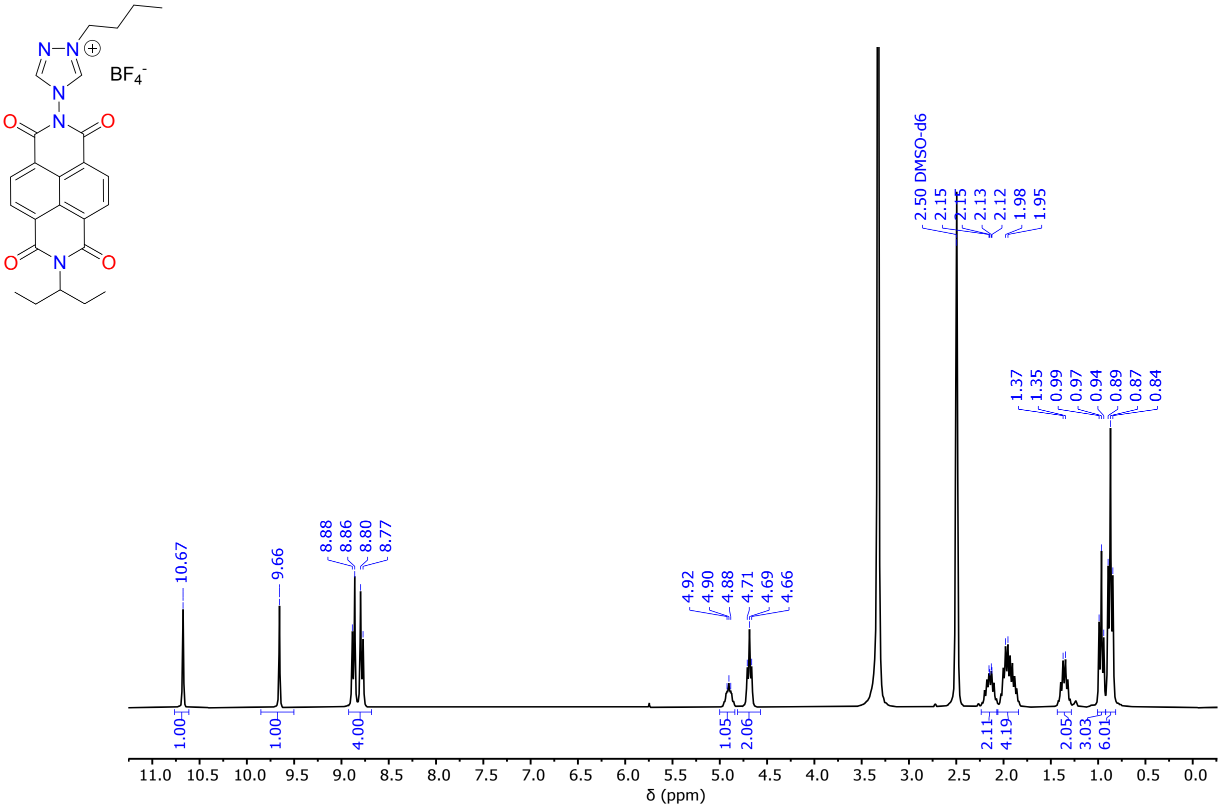

Figure S5. ^{1}H NMR spectrum (300 MHz, DMSO-d_6) of [**1**](BF_4)

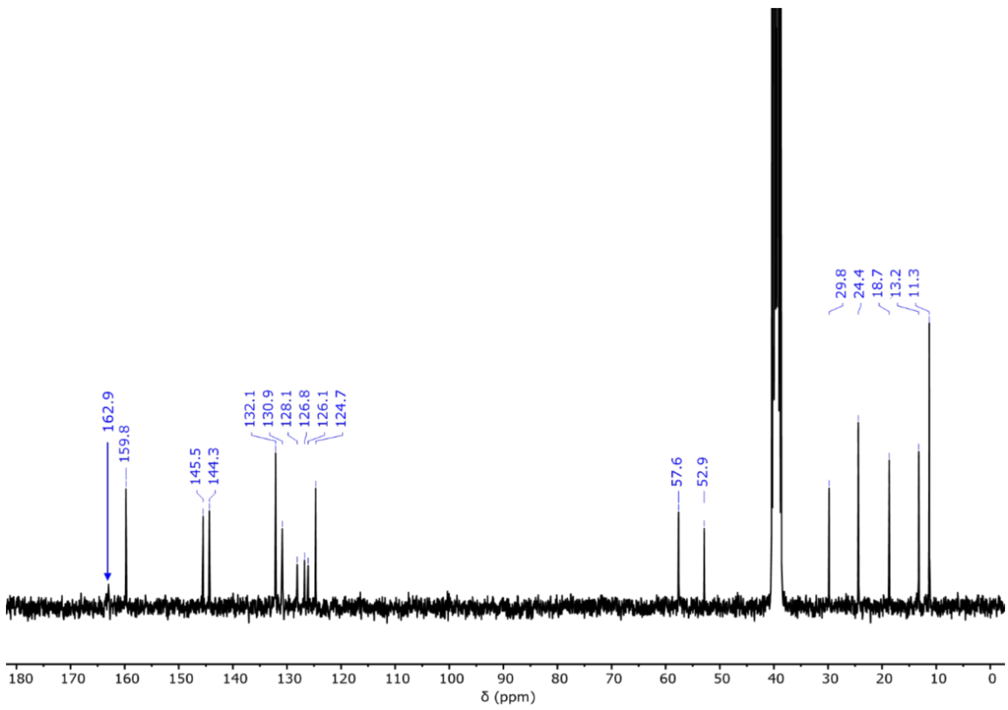

Figure S6. $^{13}C\{^{1}H\}$ spectrum (75 MHz, DMSO-d_6) of [**1**](BF_4)

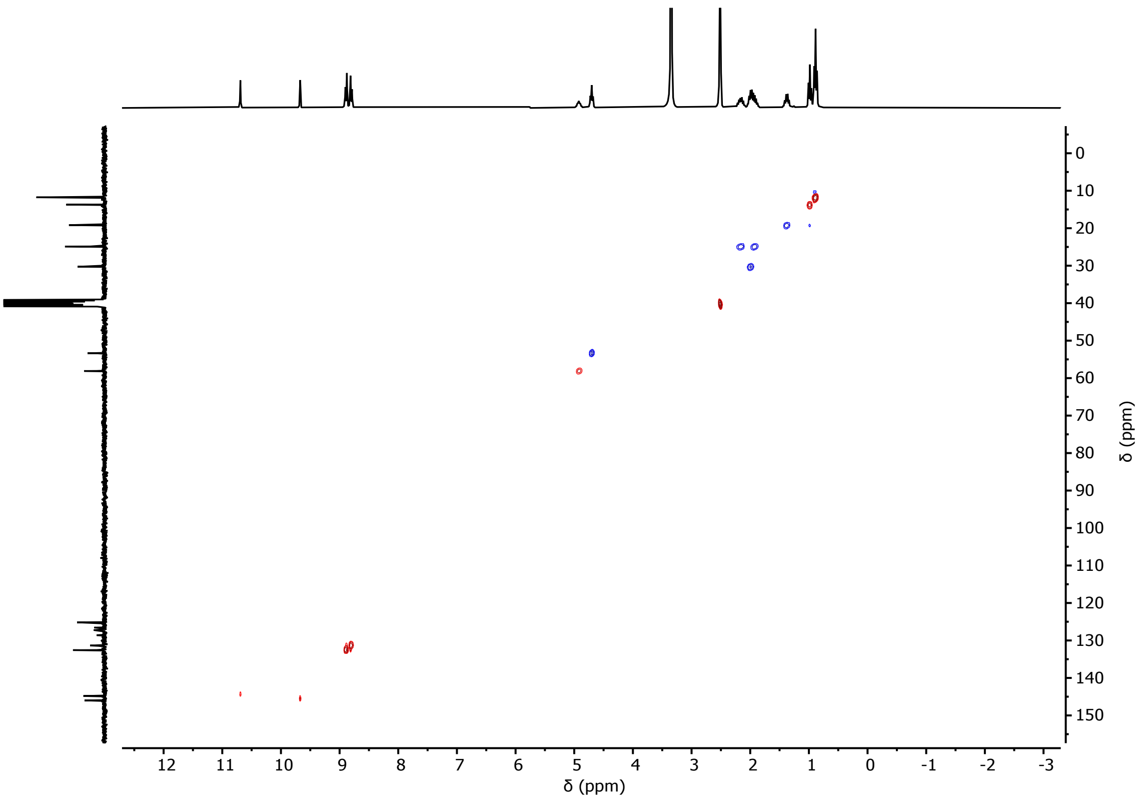

Figure S7. ^{1}H-^{13}C HSQC-NMR spectrum (300 MHz, $CDCl_3$) of [**1**](BF_4)

1.4. ^{1}H, ^{13}C{^{1}H} and HSQC NMR spectra of 2

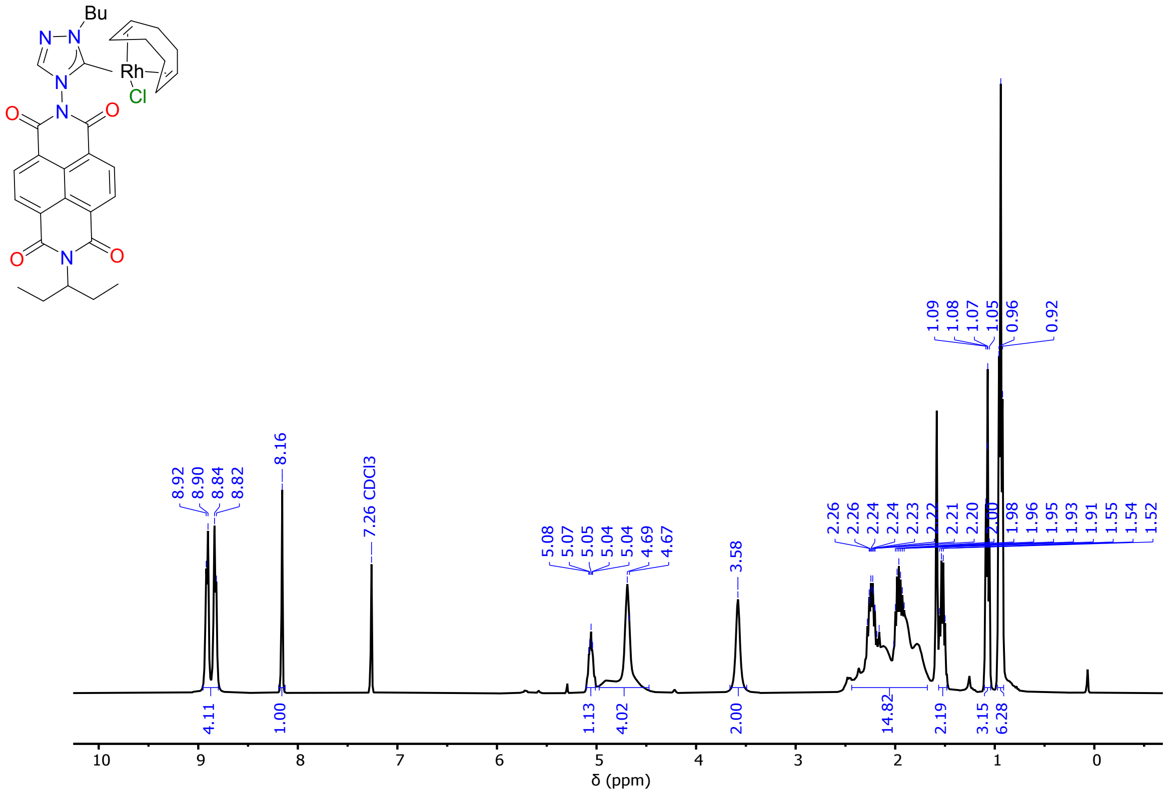

Figure S8a. ^{1}H NMR spectrum (400 MHz, $CDCl_3$) of **2**

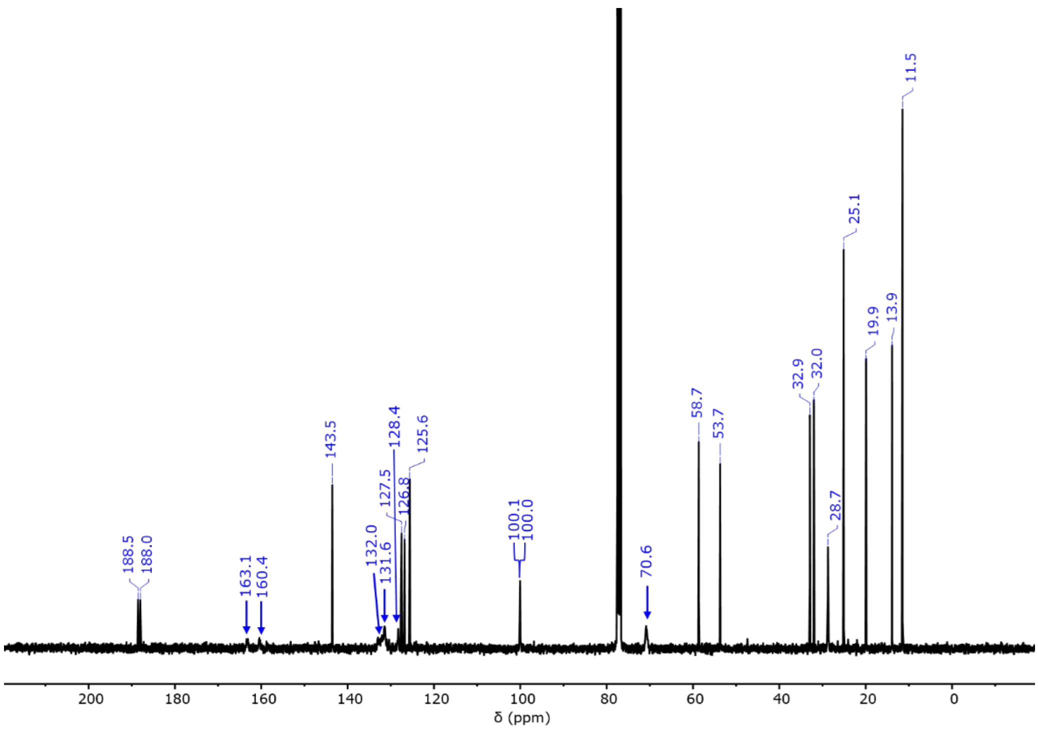

Figure S9. $^{13}C\{^{1}H\}$ spectrum (101 MHz, $CDCl_3$) of **2**

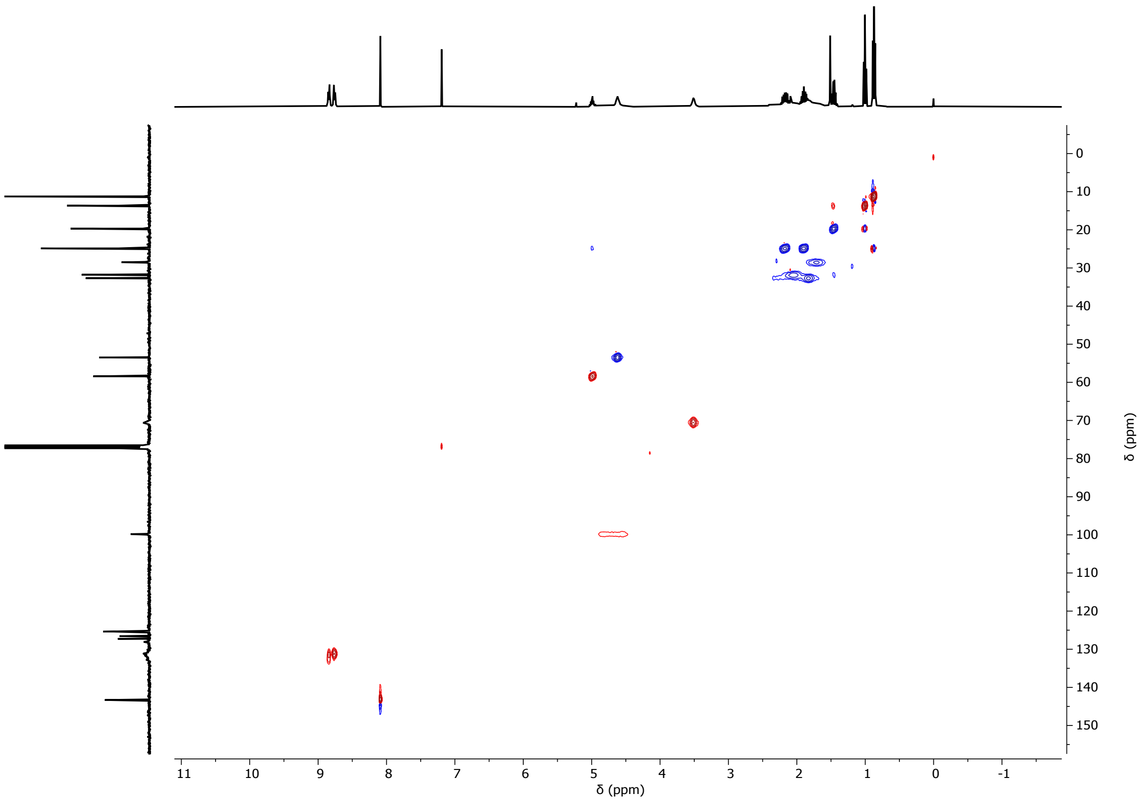

Figure S10. ^{1}H-^{13}C HSQC-NMR spectrum (400 MHz, $CDCl_3$) of **2**

1.5. ^{1}H, ^{13}C{^{1}H} and HSQC NMR spectra of 3

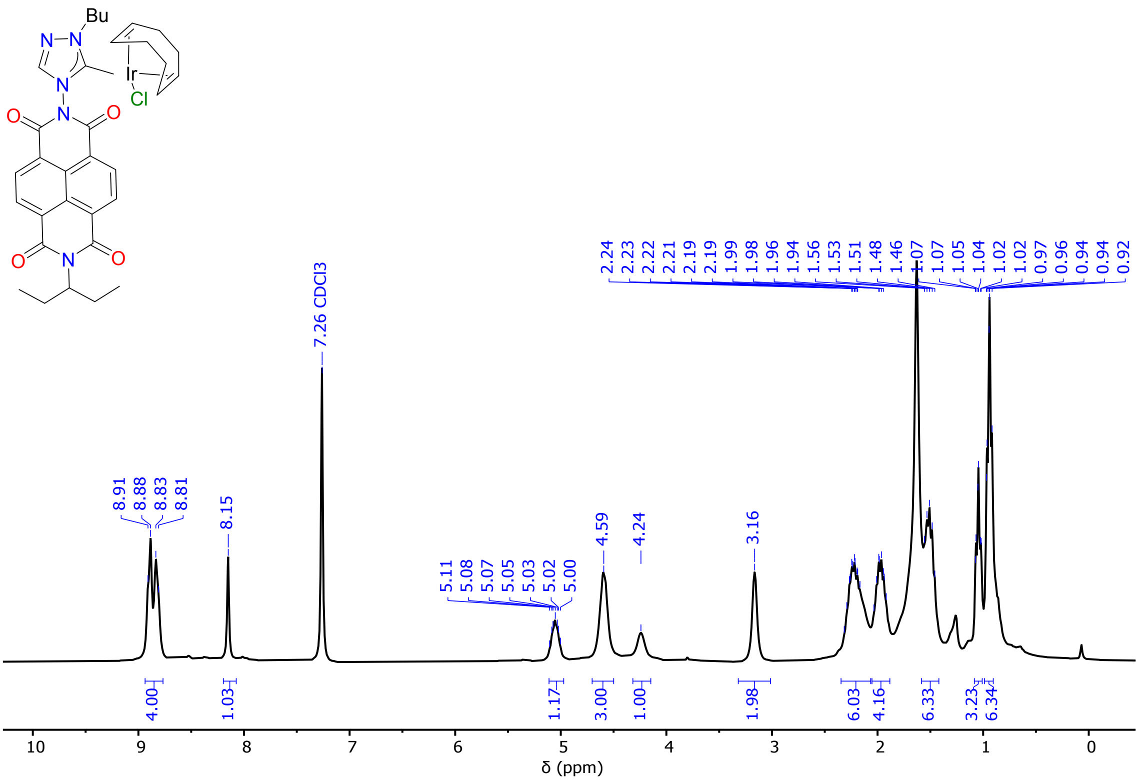

Figure S11a. ^{1}H NMR spectrum (300 MHz, $CDCl_3$) of **3**

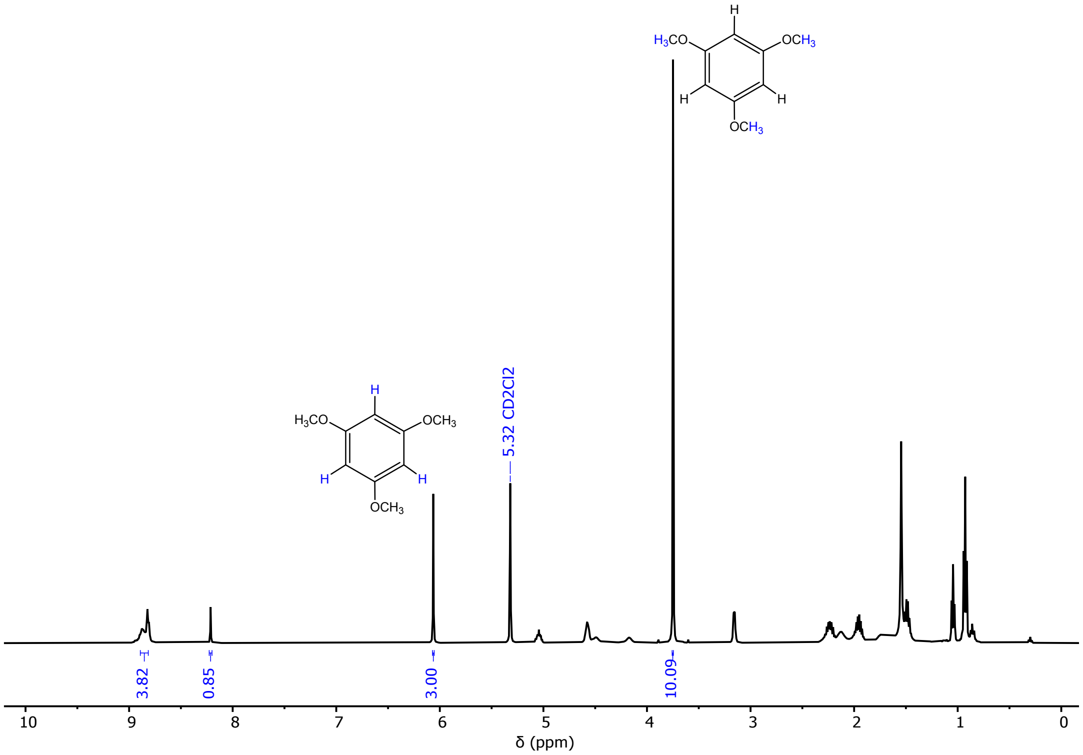

Figure S11b. ^{1}H NMR spectrum (300 MHz, CD_2Cl_2) of **3** in the presence of an equimolar amount of 1,3,5-trimethoxybenzene (purity of the bulk solid = 85%)

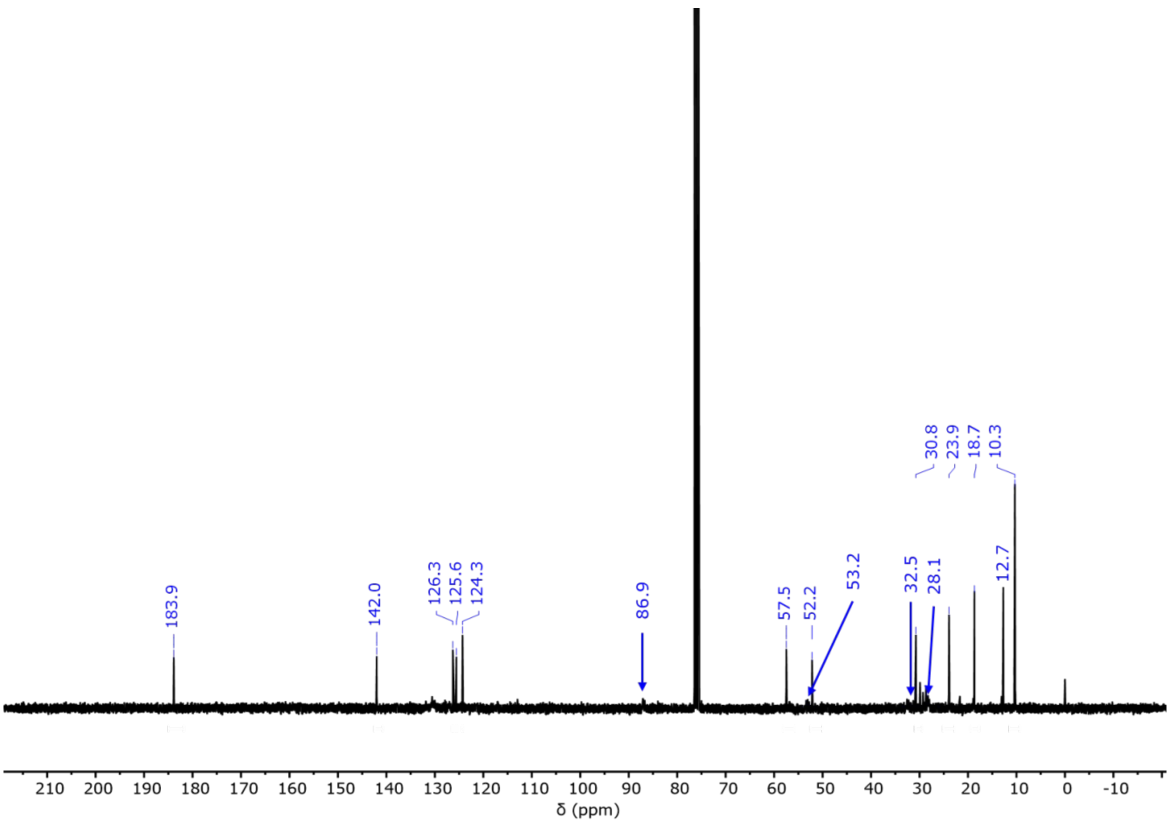

Figure S12. $^{13}C\{^{1}H\}$ spectrum (75 MHz, $CDCl_3$) of **3**

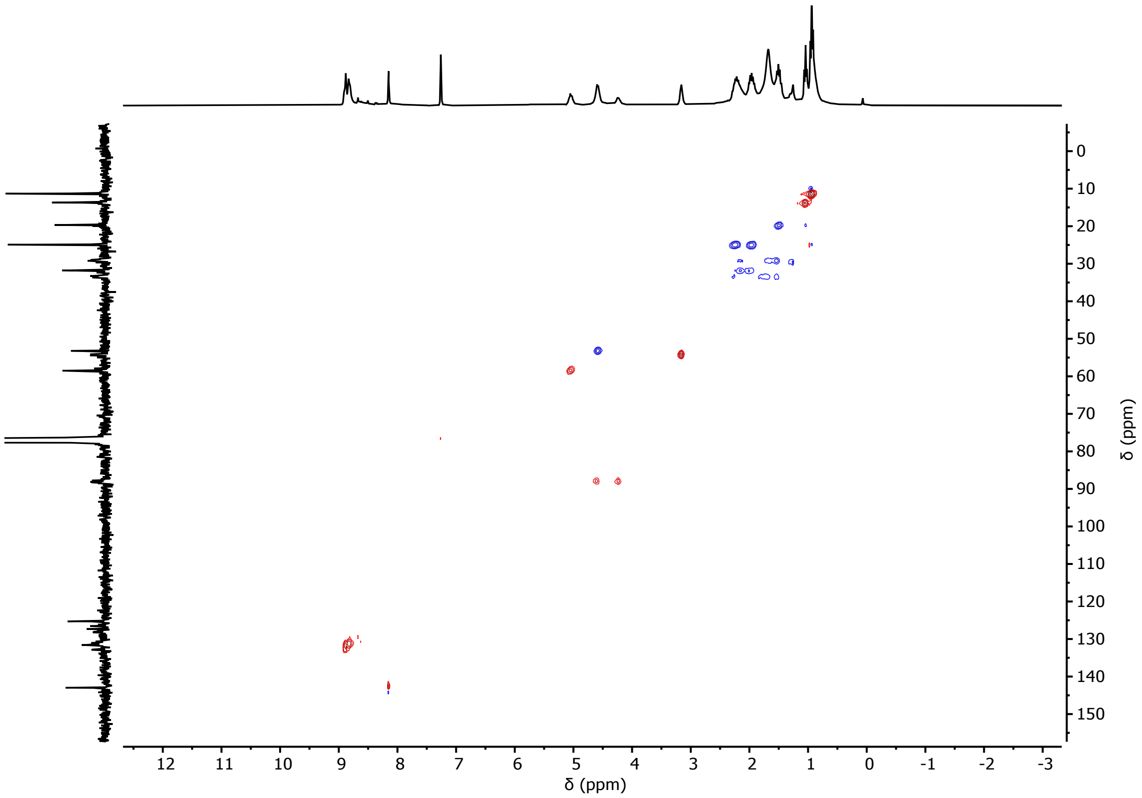

Figure S13. ^{1}H-^{13}C HSQC-NMR spectrum (300 MHz, $CDCl_3$) of **3**

1.6. ^{1}H and $^{13}C\{^{1}H\}$ NMR spectra of 4

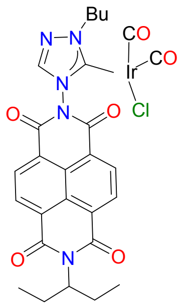

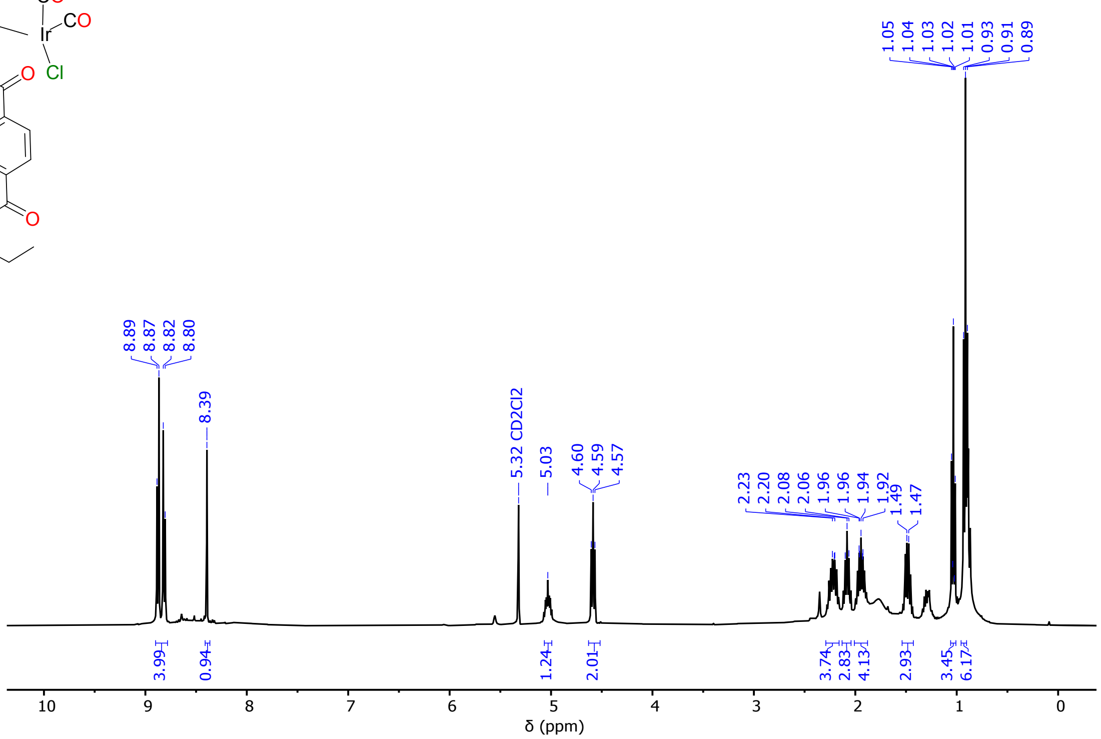

Figure S14. ^{1}H NMR spectrum (400 MHz, CD_2Cl_2) of **4**

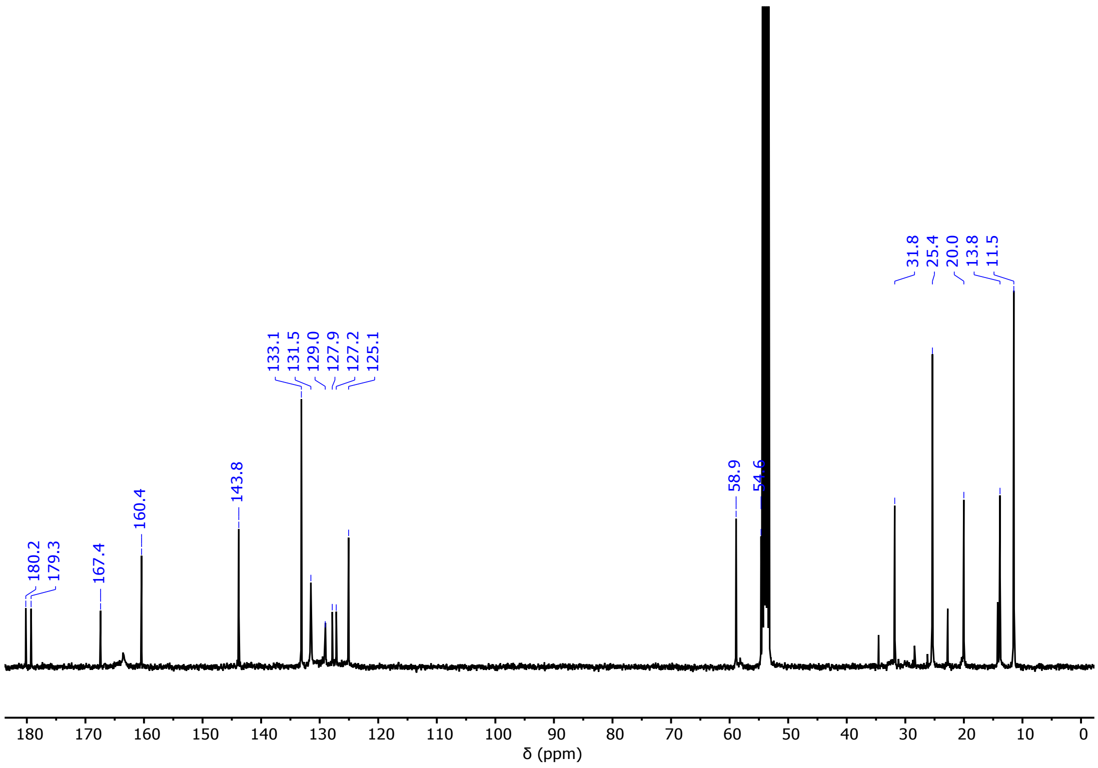

Figure S15. $^{13}C\{^{1}H\}$ spectrum (101 MHz, CD_2Cl_2) of **4**

2. HRMS spectra of the compounds

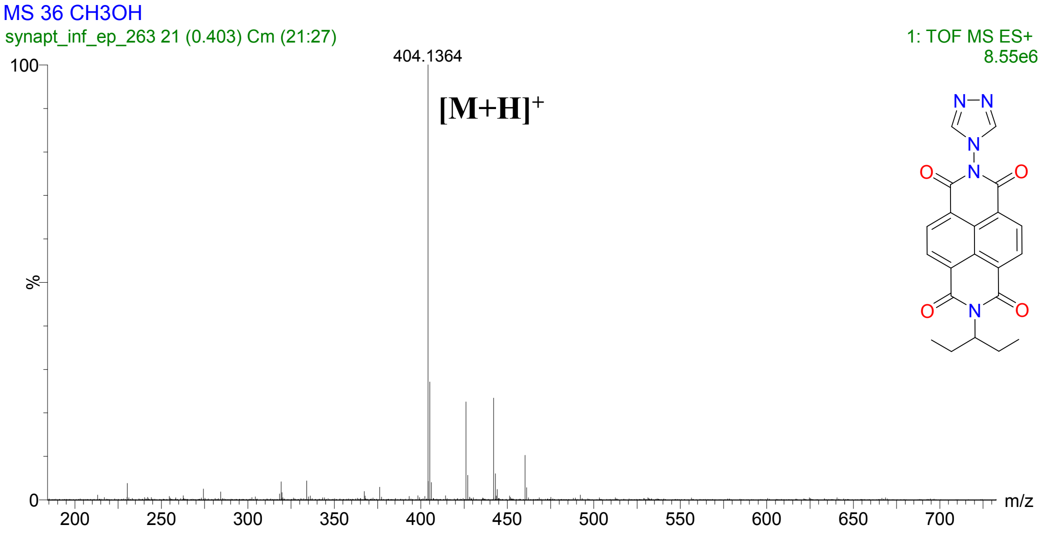

Figure S16. HRMS spectrum of **I**

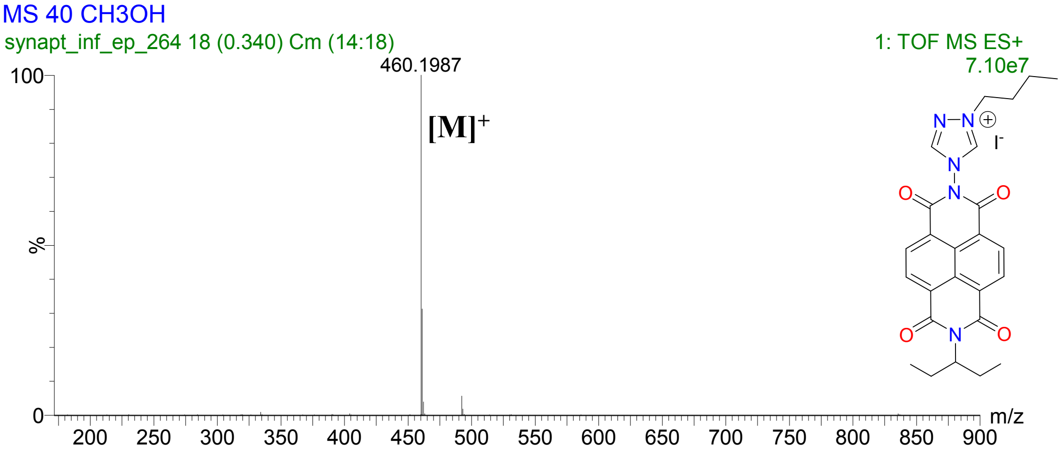

Figure S17. HRMS spectrum of [**1**](I)

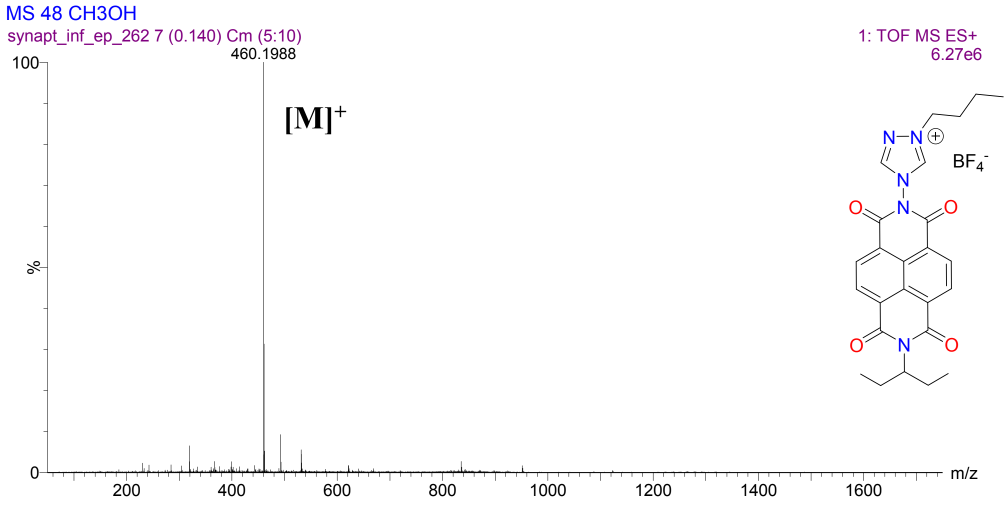

Figure S18. HRMS spectrum of [**1**](BF_4)

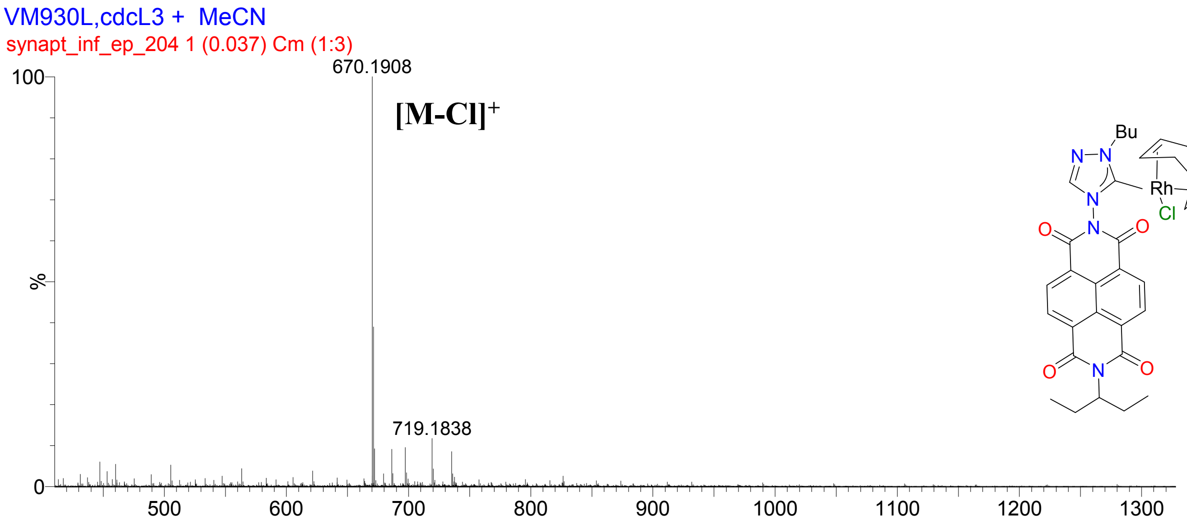

Figure S19. HRMS spectrum of **2**

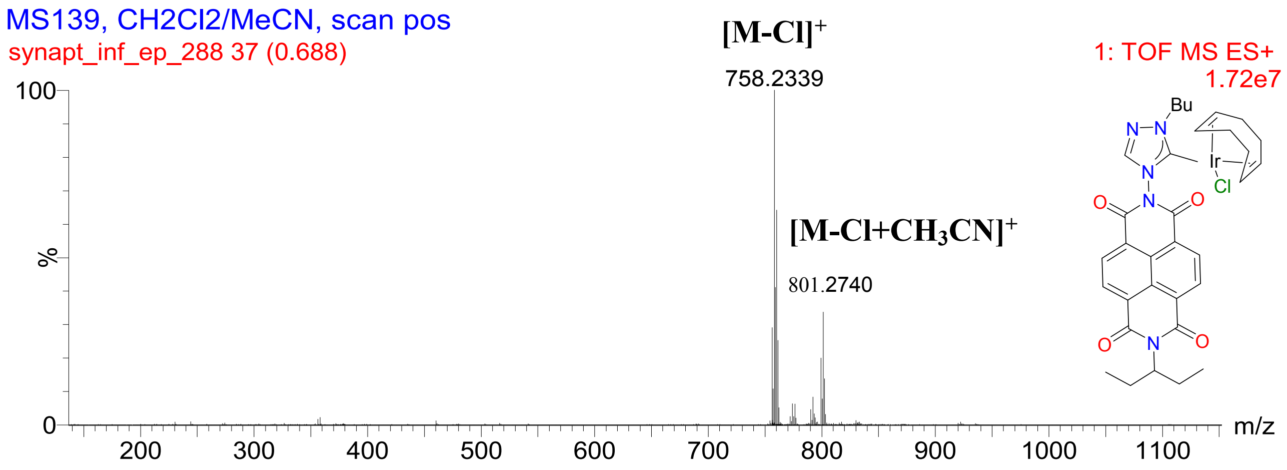

Figure S20. HRMS spectrum of **3**

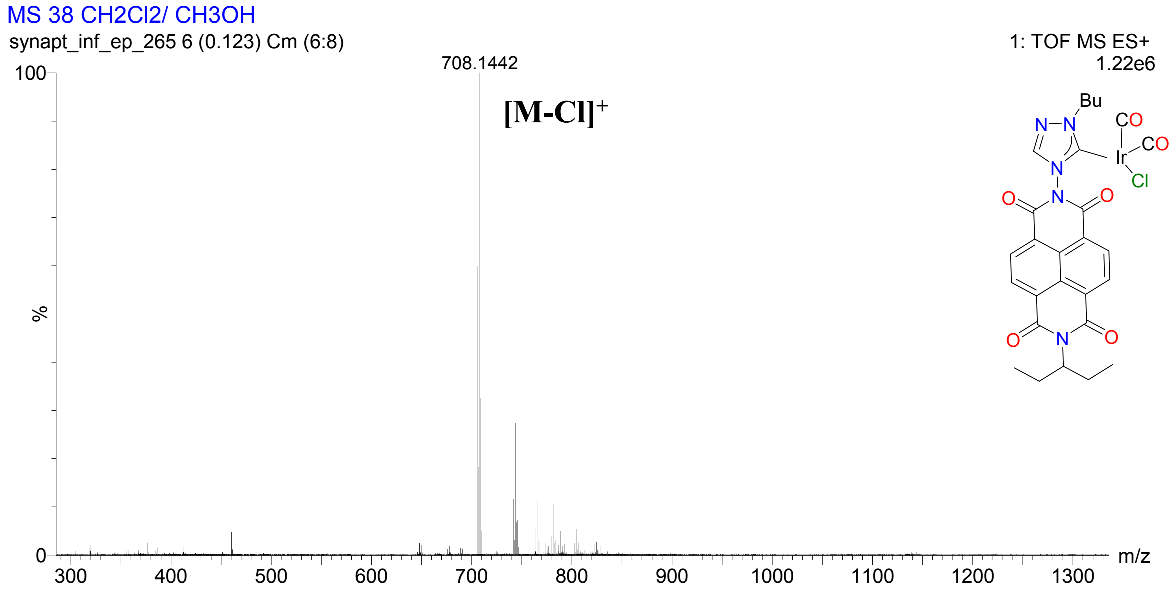

Figure S21. HRMS spectrum of **4**

3. X-ray crystallography

Single crystals for X-Ray studies of **2** were obtained by slow diffusion of tert-butyl methyl ether into a solution of the compound in dichloromethane. The diffraction data were collected on an Agilent SuperNova diffractometer equipped with an Atlas CCD detector using Cu-Kα radiation (λ = 1.54184 Å). Single crystals were mounted on a MicroMount® polymer tip (MiteGen) in a random orientation. Absorption corrections based on Gaussian methods.

Using Olex2,[1] the molecular structure of **2** was solved by Charge Flipping in Superflip,[2] and refined by least squares with the ShelXL refinement package.[3] H-atoms were introduced in calculated positions and refined on a riding model. Non-hydrogen atoms were refined anisotropically. Key details of the crystals and structure refinement data are summarized in Supplementary Table S1. Further crystallographic details may be found in the CIF files, which were deposited at the Cambridge Crystallographic Data Centre, Cambridge, UK. The reference number for complex **2** was assigned as 2486280.

Table S1. Summary of crystal data, data collection, and structure refinement details of complex **2**.

	**2**
**Empirical formula**	$C_{33}H_{37}ClN_5O_4Rh$
**Formula weight**	706.03
**Temperature/K**	200(2)
**Crystal system**	triclinic
**Space group**	P-1
**a/Å**	13.46653(10)
**b/Å**	15.33046(14)
**c/Å**	15.81969(12)
**α/°**	78.1913(7)
**β/°**	88.9667(6)
**γ/°**	78.0990(7)
**Volume/Å^3**	3127.12(5)
**Z**	4
**ρ_{calc} g/cm^3**	1.500
**μ/mm^{-1}**	5.579
**F(000)**	1456.0
**Crystal size/mm^3**	0.524 × 0.289 × 0.256
**2Θ range for data collection/°**	7.4 to 133.2
**Index ranges**	$-16 \leq h \leq 15$, $-18 \leq k \leq 18$, $-18 \leq l \leq 18$
**Reflections collected**	55460
**Independent reflections**	11041 [R_{int} = 0.0379, R_{sigma} = 0.0286]
**Data/restraints/parameters**	11041/0/799
**Goodness-of-fit on F^2**	1.044
**Final R indexes [I>=2σ (I)]**	R_1 = 0.0413, wR_2 = 0.1090
**Final R indexes [all data]**	R_1 = 0.0419, wR_2 = 0.1100
**Largest diff. peak/hole / e Å^{-3}**	1.45/-0.75

4. Electrochemical studies

4.1. Electrochemical measurements

Electrochemical studies were carried out by using an Autolab Potentiostat, Model PGSTAT101 controlled with NOVA 2.1.5 software. In all experiments, $[N(nBu)_4][PF_6]$ (0.250 M in dry and deoxygenated dichloromethane) was used as the supporting electrolyte with an analyte concentration of 1 mM. Cyclic voltammetry was performed in a cell, under N_2 atmosphere and with disk glassy carbon working electrode, platinum counter electrode, and a silver wire pseudoreference electrode. All scans were referenced to the ferrocenium/ferrocene (Fc^+/Fc) couple at 0 V. Ohmic drop was minimized by minimizing the distance between the working and reference electrodes. The residual ohmic drop was estimated by positive feedback and compensated at 95 %.

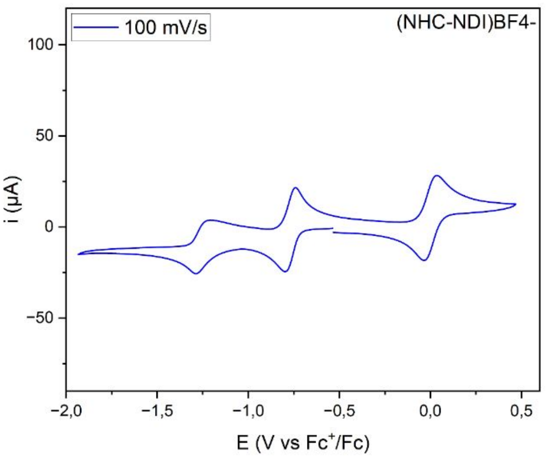

Figure S22. Cyclic voltammogram of [**1**](BF_4) at 100 mV/s.

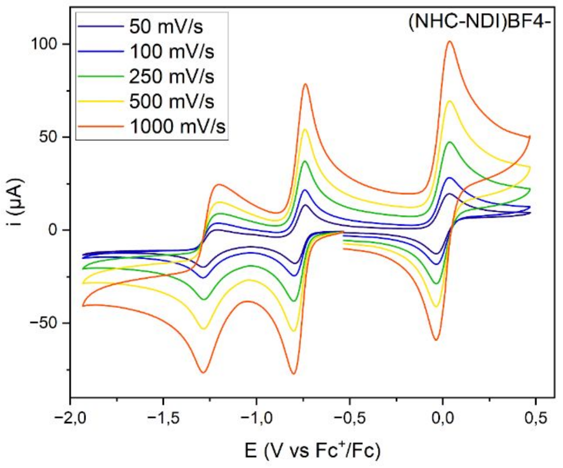

Figure S23. Cyclic voltammogram of [**1**](BF_4) at different scan rates.

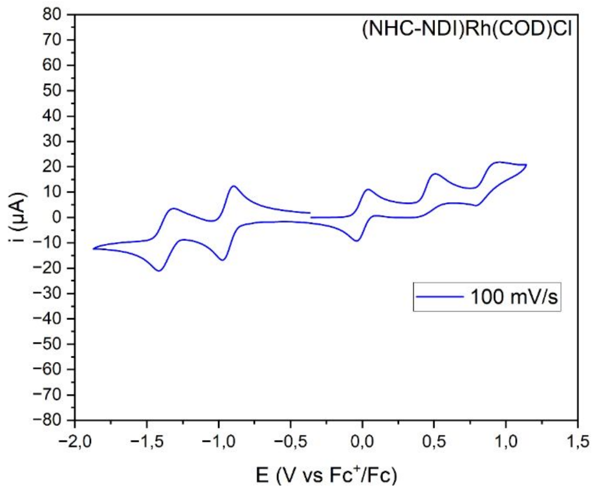

Figure S24. Cyclic voltammogram of **2** at 100 mV/s.

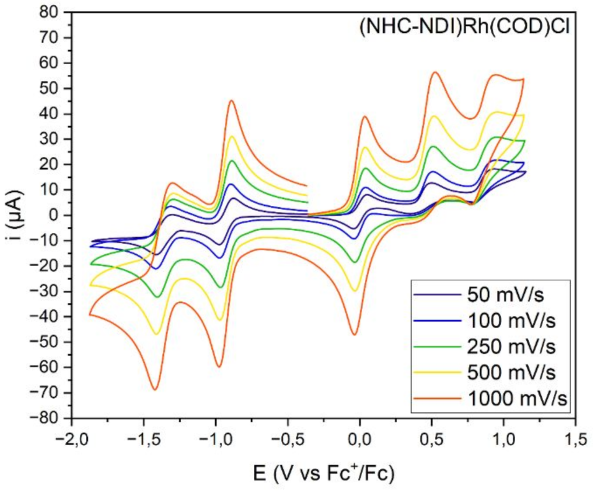

Figure S25. Cyclic voltammogram of **2** at different scan rates.

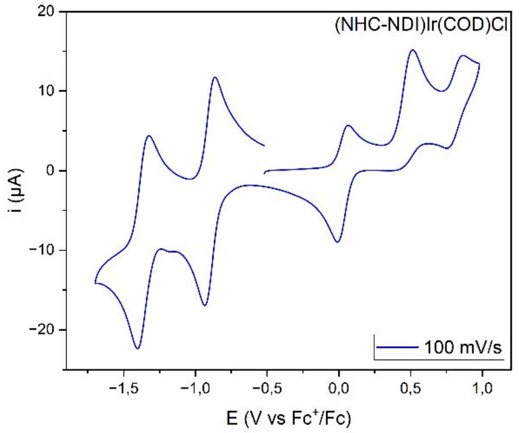

Figure S26. Cyclic voltammogram of **3** at 100 mV/s.

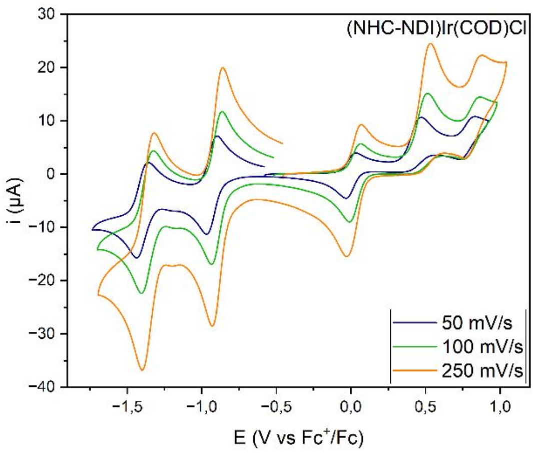

Figure S27. Cyclic voltammogram of **3** at different scan rates.

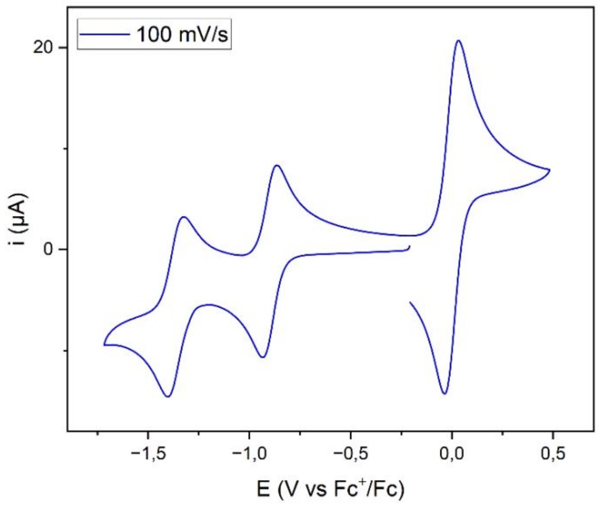

Figure S28. Cyclic voltammogram of **4** at 100 mV/s.

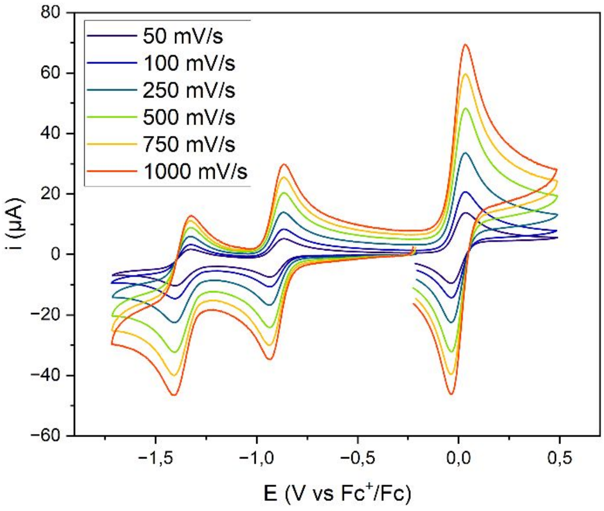

Figure S29. Cyclic voltammogram of **4** at different scan rates.

Table S2. Electrochemical properties.[a]

Compound	$E_{1/2}$ (V)/ΔE (mV)	$E'_{1/2}$ (V)/ΔE (mV)	E_{pc} (V)
[**1**](BF_4)	-0.77 / 60	-1.26 / 75	--
**2**	-0.93 / 71	-1.36 / 85	0.44
**3**	-0.90 / 65	-1.37/ 77	0.46
**4**	-0.90 / 75	-1.36/ 81	--

[a]Cyclic voltammograms performed in dry CH_2Cl_2 with 1 mM analyte and 0.250 M [N(*n*Bu)$_4$][PF_6]. Measurements performed at 100 mVs^{-1} and referenced *vs* ferrocenium/ferrocene.

4.2. Spectroelectrochemical studies

Spectroelectrochemical (SEC) measurements were performed using a gastight, optically transparent thin-layer solution cell fabricated by Prof. Hartl at the University of Reading (Reading, U.K.), as described previously.[4] The SEC cell contained a masked Pt-minigrid working electrode (32 wires/cm), a Pt-gauze auxiliary electrode and an Ag-wire pseudo-reference electrode and had CaF_2 windows. In each experiment, electrochemical reduction of the species of interest ([Analyte] = 5 mM for IR experiments and 1 mM for UV-vis experiments, [$TBAPF_6$] = 250 mM in dry CH_2Cl_2 under inert atmosphere) was monitored by the appropriate spectroscopy for a period of 2−5 min. First, the potential of the cell was swept negatively starting at the open circuit potential, recording a thin-layer cyclic voltammogram (5 mV/s) to identify the potential window of interest. Then, fresh analyte solution was introduced in the cell and the potential was varied within range of interest in 33 mV steps. The electrolysis step did not exceed 30 s. After each step an IR spectrum was collected. Diffusion and mixing of the redox products generated at the working and auxiliary electrodes in the cell were reasonably suppressed within the total experimental time (no more than 5 min for one complete measurement).

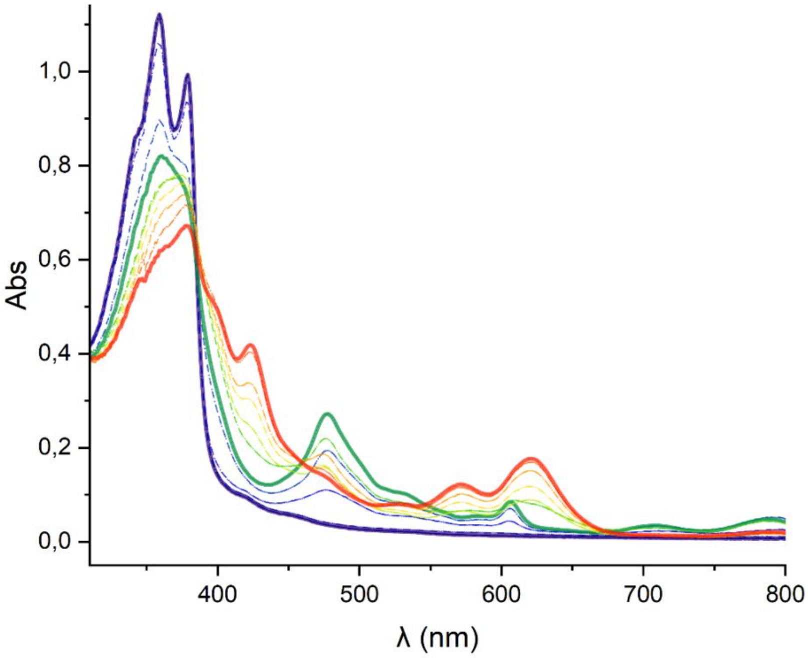

Figure S30. UV-Vis-SEC reduction of **3** in dry CH_2Cl_2 (250 mM $[N(nBu)_4][PF_6]$). The solid lines represent the UV-Vis spectra of complex **3** (blue), one-electron reduced species (green) and two-electron reduced species (red).

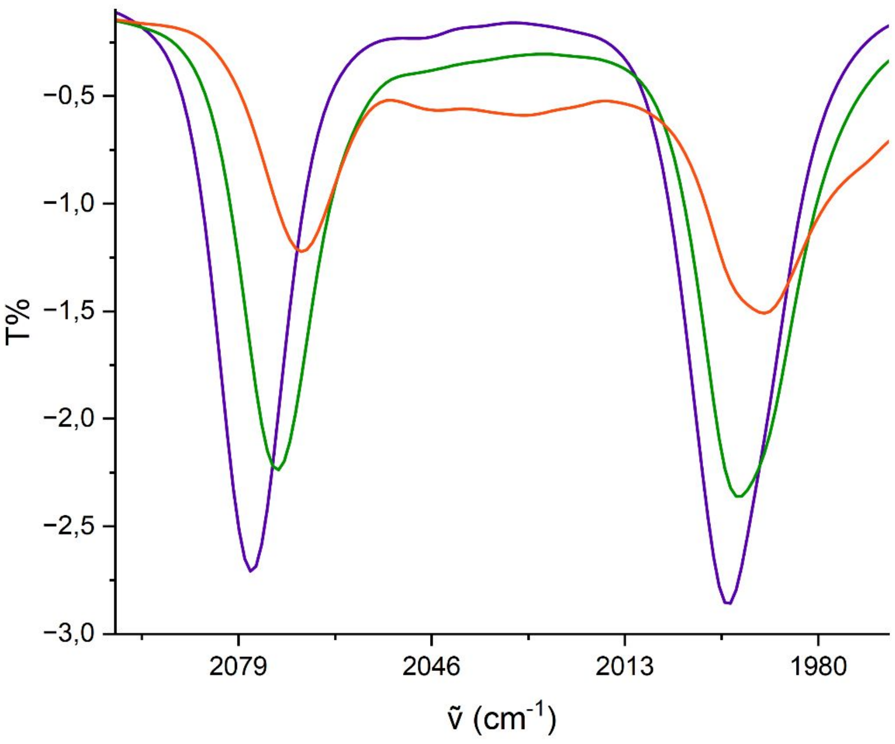

Figure S31. IR-SEC reduction of **4** in dry CH_2Cl_2 (250 mM $[N(nBu)_4][PF_6]$). The solid lines represent the IR spectra of complex **4** (blue), one-electron reduced species (green) and two-electron reduced species (red).

Table S3. CO stretching frequencies of complex **4** upon sequential reduction.

species	ν (Ir-CO) cm^{-1}	ν (Ir-CO) cm^{-1}
**4**	2077	1995
**[4˙]**	2072	1993
**$[4]^{2-}$**	2068	1988

5. Chemical reduction of complexes 2 and 3 with cobaltocene

^{1}H NMR experiments were performed to determine the effect of the addition of cobaltocene on the resonances of the starting complexes **2** and **3**. For complex **2**, an NMR tube was charged with the complex (5.3 mg, 7.5x10^{-3} mmol), 1,3,5-trimethoxybenzene (1.3 mg, 7.5x10^{-3} mmol) and 0.6 mL of CD_2Cl_2. For complex **3**, an NMR tube was charged with the complex (5.0 mg, 6.3x10^{-3} mmol), 1,3,5-trimethoxybenzene (1.3 mg, 7.5x10^{-3} mmol) and 0.6 mL of THF ($CDCl_3$ in a coaxial NMR tube was used as deuterium lock). The addition of one equivalent of cobaltocene resulted in the disappearance of the resonances due to the protons of the NDI-NHC ligand, thus indicating the formation of a paramagnetic species.

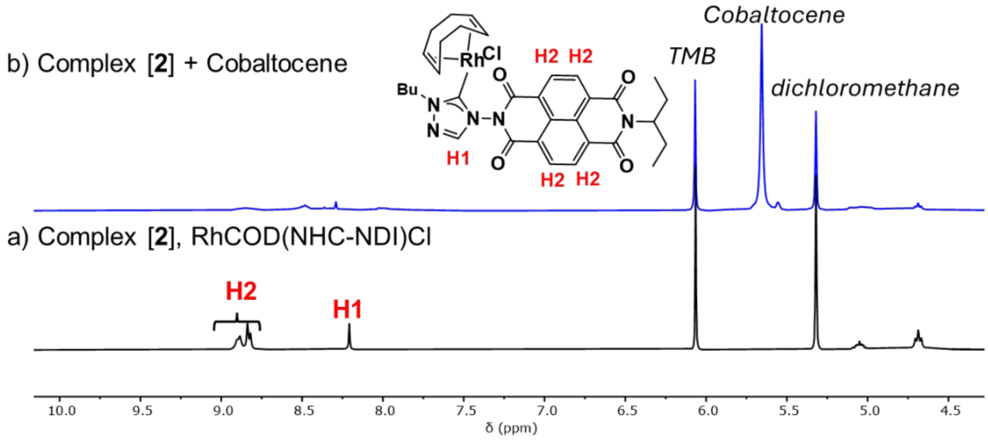

Figure S32. Selected region of the ^{1}H NMR spectra (400 MHz, CD_2Cl_2, 298 K) of **2** before (a) and after the addition of cobaltocene (b). TMB refers to 1,3,5-trimethoxybenzene, which was employed as internal standard.

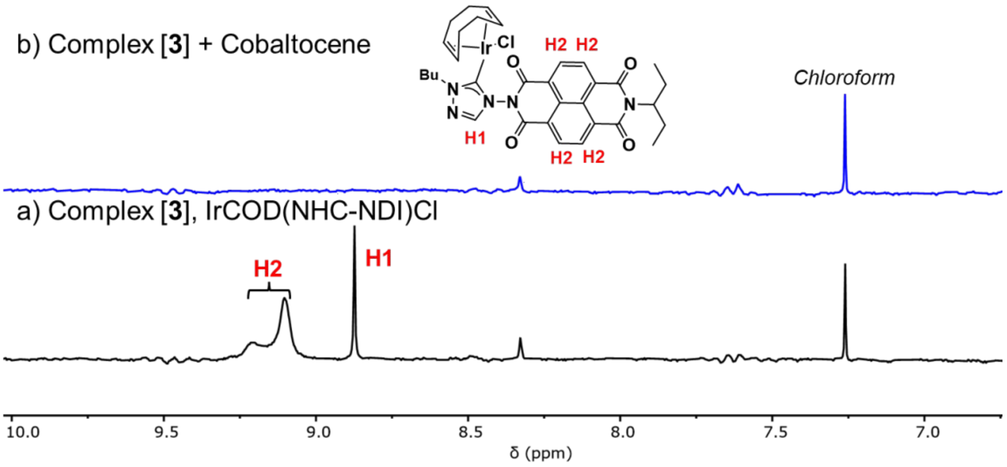

Figure S33. Selected region of the ^{1}H NMR spectra (500 MHz, THF, 298 K) of **3** before (a) and after the addition of cobaltocene (b).

6. Catalytic studies

6.1. N-methylation of primary amines using methanol

General procedure. All catalytic experiments and manipulations were conducted under nitrogen atmosphere. In a high-pressure Schlenk tube, the corresponding primary amine (0.5 mmol) reacted with an excess of methanol (1.5 mL) in the presence of cesium carbonate (0.5 equivalents, 82 mg, 0.25 mmol) and 1,3,5-trimethoxybenzene (0.5 equivalents, 42 mg, 0.25 mmol) as internal standard. The catalyst was then added at 0.5 mol % (for iridium complexes **3** and **4**) or 1 mol % (for rhodium complex **2**) catalyst loading. The resulting mixture was heated at 150 °C.

The progress of the reactions, yields, and conversions were determined by GC analysis and ^{1}H NMR spectroscopy by comparing the signals of the starting primary amine and the final product with those of the internal standard. For GC analysis, 50 µL aliquots of the reaction mixture were taken at selected time intervals and diluted in 1.5 mL of HPLC-grade toluene. For NMR measurements, 70 µL aliquots of the reaction mixture were taken at selected time intervals and diluted in 0.4 mL of $CDCl_3$.

Table S4 provides a summary of the catalytic experiment results obtained in the N-methylation of aniline with methanol according to the general procedure using complexes **2**, **3** and **4** as catalysts. For comparative purposes, additional Ir-based complexes were included in this study, namely the dimers $[IrCl(COD)]_2$ and $[IrCl_2Cp^*]_2$, as well as the NHC-based complexes **5** and **6** previously synthetized in our laboratory.

The substrate scope (Table S5) was studied employing complex **3** as the catalyst (0.5 mol % loading) with 0.5 mmol of the following substates: aniline, 4-nitroaniline, 4-methylaniline, 4-fluoroaniline, cyclohexylamine and 3-pentamine.

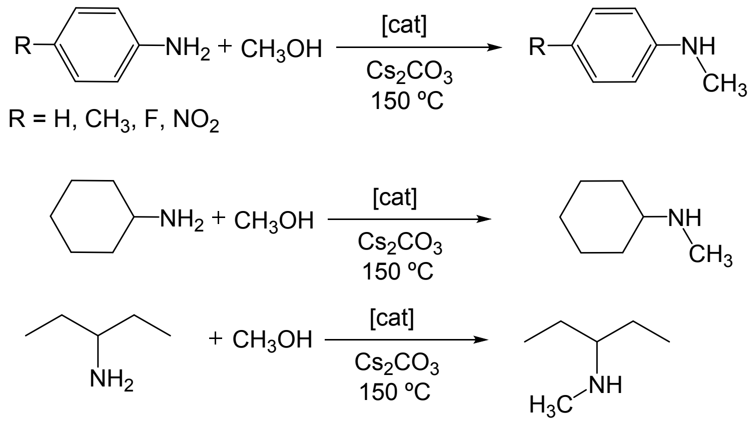

Scheme S2. N-methylation of primary amines with methanol.

Table S4. Iridium-catalyzed N-methylation of aniline with methanol.

Entry	Catalyst	Time (min)	Yield (%)
1[a]	**2**	240	5
2	**3**	60	51
3	**3**	120	89
4	**3** + 0.6 mol% [$CoCp_2$]	60	5
5	**3** + 0.6 mol% [$CoCp_2$]	120	7
6	**4**	60	30
7	**4**	120	60
8		60	9
9	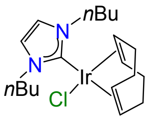 **5**	180	34
10		300	48
11		60	4
12	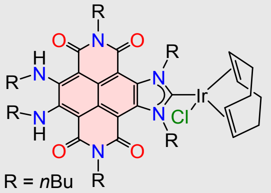 **6**	120	12
13		300	18
14		60	4
15	[$IrCl_2Cp^*$]$_2$	180	32
16		300	49
17		60	8
18	[IrCl(COD)]$_2$	180	18
19		300	25

[a]Catalyst loading = 1 mol %.

Table S5. N-methylation of primary amines with methanol using **3** as catalyst.

Entry	substrate	Time (min)	Yield (%)
1	4-methylaniline	60	42
2		120	77
3	4-nitroaniline	60	87
4		120	99
5	4-fluoroaniline	60	48
6		120	75
7	cyclohexylamine	60	2
8		180	20

9		300	28
10		60	10
11	3-pentamine	180	21
12		300	30

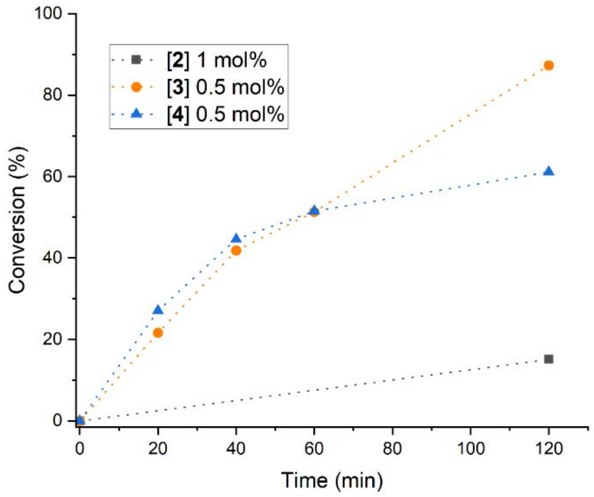

Figure S34. Time-dependent reaction profiles for the N-methylation of aniline with methanol using complexes **2**, **3** and **4**.

6.2. Determination of the reaction order with respect to catalyst 3

These experiments were conducted following the general procedure using aniline as substrate. The reaction order with respect to catalyst **3** was determined by plotting the concentration of the product against a normalized time scale t[cat]^n (being "n" the order of the catalyst), according to the method developed by Dr. Burés.[5] Visual analysis of the reaction profiles depicted in Figure S35 indicated that the order in the catalyst is 1 (Figure S35b).

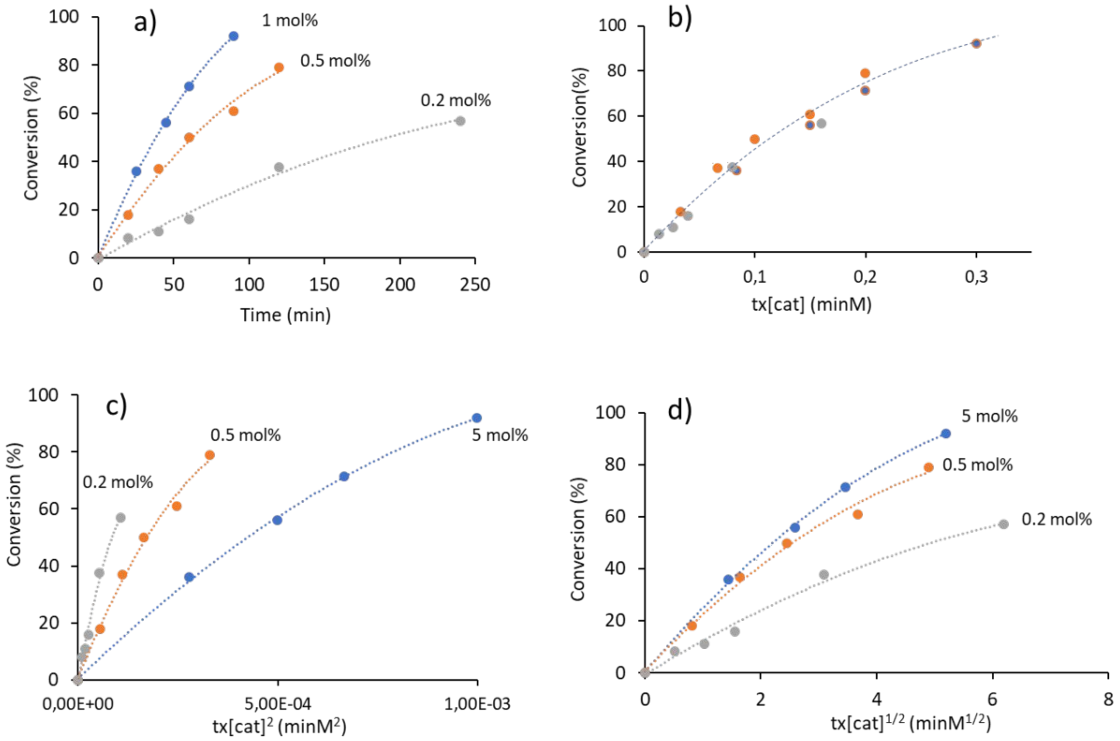

Figure S35. (a) Time-dependent reaction profile of the N-methylation of aniline using **3**. (b) Reaction profile with normalized time scale assuming a first-order order in **3**. (c) Reaction profile with normalized time scale assuming a second order in **3**. (d) Reaction profile with normalized time scale assuming half-order order in **3**.

6.3. Redox switching experiments

The redox switching experiments were carried out following the general procedure using aniline as substrate and catalyst **3**. Aniline (45.5 µL, 0.5 mmol), methanol (1.5 mL), cesium carbonate (0.5 equivalents, 82 mg, 0.25 mmol) and 1,3,5-trimethoxylbenzene (0.5 equivalents, 42 mg, 0.25 mmol) as internal standard, were placed together in a high-pressure Schlenk tube. Complex **3** (0.5 mol%) and the reductant cobaltocene (1.2 equivalents with respect to the catalyst) were subsequently added under nitrogen, and the resulting reaction mixture was heated at 150ºC. After monitoring the reaction for 60

minutes, acetylferrocenium tetrafluoroborate was added as oxidizing agent (1.2 equivalent with respect to the catalyst). After monitoring the reaction for 60 minutes, cobaltocene was added again to the reaction (1.2 equivalents with respect to the catalyst). The evolution of the reaction, yields, and conversions were determined by GC analysis comparing the areas of aniline with those of the internal standard. Blank tests in the presence of either cobaltocene or ferrocenium tetrafluoroborate were also run to rule out any catalytic activity of the redox additives.

7. References

1. Dolomanov, O. V.; Bourhis, L. J.; Gildea, R. J.; Howard, J. A. K.; Puschmann, H., OLEX2: *A complete structure solution, refinement and analysis program*. *J. Appl. Crystallogr.* **2009,** *42*, 339-341.

2. Palatinus, L.; Chapuis, G., SUPERFLIP - a computer program for the solution of crystal structures by charge flipping in arbitrary dimensions. *J. Appl. Crystallogr.* **2007,** *40*, 786-790.

3. Sheldrick, G. M., SHELXT - Integrated space-group and crystal-structure determination. *Acta Crystallogr. A* **2015,** *71*, 3-8.

4. Krejcik, M.; Danek, M.; Hartl, F., Simple Construction Of An Infrared Optically Transparent Thin-Layer Electrochemical-Cell - Applications To The Redox Reactions Of Ferrocene, Mn2(Co)10 And Mn(Co)3(3,5-Di-Tert-Butyl-Catecholate). *J. Electroanal. Chem.* **1991,** *317*, 179-187.

5. (a) Bures, J., A Simple Graphical Method to Determine the Order in Catalyst. *Angew. Chem. Int. Ed.* **2016,** *55*, 2028-2031; (b) Bures, J., Variable Time Normalization Analysis: General Graphical Elucidation of Reaction Orders from Concentration Profiles. *Angew. Chem., Int. Ed.* **2016,** *55*, 16084-16087.
